# Supplementary material for: The Genomic-Driven Discovery of Glutarimide-Containing Derivatives from Burkholderia gladioli
Source: Molecules. 2023 Oct 5;28(19):6937. doi: 10.3390/molecules28196937 (PMC10574677; doi:10.3390/molecules28196937)
Supplement: Supplementary file 1 [file molecules-28-06937-s001.zip › molecules-2568878-supplementary.pdf]

Supplementary DATA

The Genomic-Driven Discovery of Glutarimide-Containing Derivatives from *Burkholderia gladioli*

**Hanna Chen [1,2,†], Xianping Bai [1,†], Tao Sun [1], Xingyan Wang [1], Youming Zhang [1,3,*], Xiaoying Bian [1,*]
and Haibo Zhou [1,*]**

[1] Helmholtz International Lab for Anti-Infectives, Shandong University–Helmholtz Institute of Biotechnology, State Key Laboratory of Microbial Technology, Shandong University, Qingdao 266237, China; chenhannahappy@163.com (H.C.); xpbai201812479@163.com (X.B.); suntao_go@126.com (T.S.); 202012620@mail.sdu.edu.cn (X.W.)

[2] School of Medicine, Linyi University, Shuangling Road, Linyi 276000, China

[3] CAS Key Laboratory of Quantitative Engineering Biology, Shenzhen Institute of Synthetic Biology, Shenzhen Institute of Advanced Technology, Chinese Academy of Sciences, Shenzhen 518055, China

[*] Correspondence: zhangyouming@sdu.edu.cn (Y.Z.); bianxiaoying@sdu.edu.cn (X.B.); haibozhou@sdu.edu.cn (H.Z.)

[†] These authors contributed equally to this work.

Materials and Methods

1. General Experimental Procedures

Optical rotations were obtained on a JASCO P-1020 digital polarimeter (JASCO Corporation, Tokyo, Japan). UV spectra were recorded on a Thermo Scientific Dionex Ultimate 3000 DAD detector. ^{1}H and ^{13}C NMR, DEPT, and 2D NMR spectra were recorded on an Agilent 600 MHz DD2 (Agilent Technologies Inc., Santa Clara, CA, USA) using residual solvent signals of $CDCl_3$ ($\delta_{H/C}$ 7.26/77.16) as the internal standard. HR-ESI-MS spectra were measured on a Bruker Impact HD microTOF Q III mass spectrometer (Bruker, Rheinstetten, Germany) using the standard ESI source. A Thermo Scientific™ Acclaim™ C_{18} column (2.1×100 mm, 2.2 μm) was used. The mobile phase consisted of H_2O and acetonitrile (ACN) both containing 0.1% formic acid (FA). The bioinformatic analysis of ATCC 10248 genome employs AntiSMASH website (https://antismash.secondarymetabolites.org/#!/start) and Pfam website (http://pfam.xfam.org/.). [1,2]

2. Bacterial Strains, Plasmids and Reagents

The strains, mutants and plasmids used in this study are list in Table S3. *B. gladioli* ATCC 10248 was ordered from the China General Microbiological Culture Collection Center (CGMCC). The two heterologous hosts *Caldimonas brevitalea* DSM 7029Δ*glb-attB* and *Burkholderia thailandensis* E264Δ*oprC-attB* were engineered by our group to express exogenous BGCs. [3,4] The procedure of direct cloning and Red/ET recombineering was performed into *Escherichia coli* GB05Dir/pSC101-BAD-ETgA-tet for linear and linear homology recombination (LLHR) and *E. coli* GB08Red for linear and circle homology recombination (LCHR). [5] Polymerase chain reaction (PCR) amplification used 2×*ApexHF* HS DNA polymerase FS Master Mix (Accurate Biotechnology (Hunan) Co., Ltd., China).

All *E. coli* strains were grown on Luria broth (LB) medium. The antibiotic concentrations for *E. coli* strains on plate or liquid of LB medium were as follows: chloramphenicol (Cm 15 or 10 μg mL^{-1}), ampicillin (Amp 100 or 50 μg mL^{-1}), gentamicin (Genta 4 or 2 μg mL^{-1}). *B. gladioli* ATCC 10248, *C. brevitalea* DSM 7029, *B. thailandensis* E264 and corresponding mutants were cultured on CYMG medium (Casein peptone 8 g L^{-1}, Yeast extract 4 g L^{-1}, $MgCl_2$ $6H_2O$ 8.66 g L^{-1}, Glycerol 5 mL L^{-1}) at 30 °C. The antibiotic concentrations for ATCC 10248 strains on plate or liquid of CYMG medium were as follows: kanamycin (Km 30 or 20 μg mL^{-1}), Genta (120 or 100 μg mL^{-1}) and apramycin (Apra 250 or 200 μg mL^{-1}). The antibiotic concentrations for DSM 7029 strains on plate or liquid of CYMG medium were as follows: Km (30 or 20 μg mL^{-1}) and Genta (25 or 20 μg mL^{-1}). The gentamicin antibiotic concentrations for E264 strains on plate or liquid of CYMG medium was 6 or 5 μg mL^{-1}. *B. gladioli* ATCC 10248 and mutants were cultured on M9 medium (Glucose 10 g L^{-1}, K_2HPO_4 7 g L^{-1}, KH_2PO_4 2 g L^{-1}, $(NH_2)_2SO_4$ 1 g L^{-1}, Sodium citrate 0.5 g L^{-1}, $MgSO_4$ $7H_2O$ 0.1 g L^{-1}) for scale-up fermentation.

3. Knockout and Promoter Insertion of the Silent trans-AT Gene Cluster on the Chromosome 2 of B. gladioli ATCC 10248

The target gene *glaC* was deleted by the gentamicin resistance gene using the Redγ-BAS system. The activation mutant was constructed by insertion of a constructive promoter (P_{Genta}) replacing the original promoter in front of the gene *glaP* of the *trans*-AT BGC. The antibiotic resistance gene and constructive promoter flanked with homology arms (50 bp) were generated by PCR amplification, and the template for $genta^R$ was derived from plasmid pFox-genta-P_{tet}-LTX, respectively. For the recombineering, purified PCR products of resistance gene were transformed into *B. gladioli* ATCC 10248/pBBR1-Rha-Redγ-BAS-km, respectively. Recombinants were selected on CYMG plates containing gentamicin antibiotic. Correct recombinants were verified by colony PCR. The knockout of gladiolin BGC used apramycin gene according to our previous study. [6] A list of recombinants generated in this study is provided in Table S3. Primers used for gene cluster modification are listed in Table S4.

4. Direct Cloning and Engineering of trans-AT BGC

The *trans*-AT BGC from *B. gladioli* ATCC 10248 was directly cloned with primers pBeloBAC-cm-10248Chr2C8-S/A by ExoCET method [7] and engineered with primers Chr2C8-10248-Pgenta-S/A or BAC-BGC8-amp-attP-S/A by LCHR, resulting in the constructs of pBeloBAC-cm-Chr2C8, pBeloBAC-cm-P_{Genta}-Chr2C8, and pBeloBAC-amp-attP-P_{Genta}-Chr2C8, respectively. The genomic DNA was digested with *Spe*I and *Xba*I for cloning the 76-kb *trans*-AT gene cluster.

The final construct pBeloBAC-amp-attP-P_{Genta}-Chr2C8 was electroporated into *S. brevitalea* DSM 7029Δ*glb-attB* strain, and the Chr2C8 BGC was integrated into the glidobactin BGC region (phiC31 attB loci) of the chromosome via site-specific integration. For heterologous expression of Chr2C8 in *B. thailandensis* E264, the final construct was introduced into the specific chromosomal site with the help of donor strain *E. coli* WM3064 [8], which is autotrophic for diaminopimelic acid (DAP). The correct transformants were verified by colony PCR using check primers.

5. HPLC-HR-MS Analysis of Crude Extracts

The procedure of fermentation and extraction were referred to the previous method. [6] The UHPLC system was performed using an ODS column (Luna RP-C18, 4.6×250 mm, 5 μm, 0.75 mL/min) with gradient elution. Mass spectra was acquired in centroid mode ranging from 100 to 1500 *m*/*z* with negative-mode electrospray ionization and auto MS^2 fragmentation. HPLC parameters were set as follows: solvent A, H_2O with 0.1% FA; solvent B, 0.1% FA in ACN; gradient at a constant flow rate of 0.75 mL/min, 0-5 min, 5% B; 5-55 min, 5%-95% B; 55.1 min, 95% B; 55.1-60 min, 95% B; 60.1 min, 5% B; 60.1-65 min, 5% B; detection by UV spectroscopy at 190-400 nm.

*6. Fermentation, Extraction, and Isolation of Compounds **1**-**7***

In order to determine the structures of seven compounds produced by activated mutant *B. gladioli* ATCC 10248Δ*gbn*P_{Genta}-Chr2C8, scale-up fermentation was carried

out. *B. gladioli* ATCC 10248Δ*gbn*P_{Genta}-Chr2C8 was inoculated to CYMG medium with gentamicin 100 μg mL^{-1} at 30°C at 200 rpm for overnight. A fraction (1%, 0.5 mL) of the overnight culture was inoculated into fresh 50 mL M9 medium in 250 mL flask for 3 days at 30°C and 200 rpm shaking in the air without avoiding light.. Then XAD 16 (2%) was added (after 3 days' incubation) in the culture and the mixture was continually cultured at 30 °C, 200 rpm for 1 day. The resin was collected by sieving, washed with ddH_2O, and then extracted twice with ethyl acetate (EtOAc), which was concentrated under reduced pressure at 30°C warmed in the water bath to obtain crude extract. Then, the crude extracts were purified by MPLC at room temperature (YMC ODS-A; 5 μm, 250×20 mm, gradient elution 0-5 min, 30% ACN; 5-65 min, 85% ACN; 65.1 min, 95% ACN; 65.1 min-75 min, 95% ACN; 8 mL/min) to afford 7 fractions. Then, Fr. 2, Fr. 4, and Fr. 5 were further purified by semipreparative HPLC at 30°C which was controlled by column oven (ODS; 5 μm, 250×10 mm, isocratic gradient elution 45% ACN, 49% ACN, and 52% ACN, respectively, 3 mL min^{-1}) to yield compound **1** (8 mg), **2** (5 mg) and **5** (5 mg).

In addition, the dried crude extracts were also isolated by normal phase silica gel column using step gradient elution with Petroleum ether and EtOAc (50:1 to 1:6) due to its instability during above process. Total 18 fractions were obtained. Fr. 12 (56 mg) was purified by semi-preparative normal-phase HPLC at 30°C controlled by column oven (COSMOSIL 5SL-IIPacked Column, 4.6×250 mm, 1 mL min^{-1}) using isopropanol [0.1% trifluoroacetic acid (TFA)] and hexane as mobile phase with the following conditions: 0−5 min, 86% hexane; 5−25 min, 40% hexane; 25−30 min, 40% hexane to yield **4** (29 mg) at the retention time 18 min. Fr. 15 was further purified with the following conditions: 0−5 min, 78% hexane; 5−36 min, 52% hexane; 36.1 min, 10% hexane; 36.1−45 min, 10% hexane to yield **7** (10 mg) at the retention time 31 min. Fr. 17 (104 mg) was purified with the following conditions: 0−5 min, 82% hexane; 5−25 min, 72% hexane; 25.1 min, 50% hexane; 25.1−30 min, 50% hexane to yield **6** (13 mg) at the retention time 15 min. The fraction (1:3) was further purified by the condition: 0−3min, 78% hexane; 3−40 min, 78%-50% hexane; 40.1 min, 10% hexane; 40.1−45min, 10% hexane to generate **3** at the retention time 15 min. The isolated compounds (**3**, **4**, **6**, and **7**) was tested by LC-MS.

7. Cytotoxic and Anti-inflammatory Activities Screening

Five cell lines containing human colon cancer cell HCT-116, human breast adenocarcinoma cells MCF7, human breast cancer cell BT20, human kidney cell HEK-293T, and mouse mononuclear macrophage leukemia cell RAW246.7 were selected for cytotoxic activity screening. The negative control was DMSO with the same concentration, and the positive control was doxorubicin (sigma). The detailed procedure was performed according to methods previously described. [9] The anti-inflammatory activity of the compounds was evaluated by measuring their inhibitory effects on nitric oxide (NO) generation in LPS-stimulated RAW 264.7 macrophages based on the classic Griess method. [10] RAW 264.7 cells were seeded in 96-well plates and incubated for 24 h. Then, cells were treated with different concentrations of

compounds for 30 min followed by LPS (10 mg/mL) stimulation for another 24 h. The NO assay kit with Griess reagents (Beyotime, Lot: S0021, Shanghai, China) was used to examine cellular supernatant nitrite accumulation, which represents cellular NO levels. The OD value of 540 nm absorbance was detected on a microplate reader.

Table S1. Putative functions of proteins encoded by genes in the gladiofungin/gladiostatin biosynthetic gene cluster

Genes/ Proteins	Length bp / aa[#]	Similar Proteins	Identity (%)
-2	720 / 240	YceH family protein (*Burkholderia gladioli*) WP_036055449.1	100%
-1	602 / 200	IS5 family transposase (*Burkholderia gladioli*) WP_186267901.1	92%
P	702 /234	Histidine phosphatase family protein (*Burkholderia gladioli*) WP_036055450.1	100%
A	3360 / 1120	ACP S-malonyltransferase (*Burkholderia gladioli*) WP_042287093.1	100%
B	243 / 81	Phosphopantetheine-binding protein (*Burkholderia gladioli*) WP_036055451.1	100%
C	1971 / 657	Asparagine synthase (*Burkholderia gladioli*) WP_198399553.1	100%
D	9753 / 3251	SDR family oxidoreductase (*Burkholderia gladioli*) WP_052710568.1	100%
E	26505 / 8835	Beta-ketoacyl-acyl-carrier-protein synthase II / SDR family NAD(P)-dependent oxidoreductase (*Burkholderia gladioli*) AJW96760.1	100%
E1	8748 / 2916	SDR family NAD(P)-dependent oxidoreductase (*Burkholderia gladioli*) WP_177682598.1	99%
F	714 / 238	HAD family hydrolase (*Candidatus Accumulibacter* sp.) MBK8384182.1 Hypothetical protein (*Burkholderia gladioli*) WP_036055454.1	63%/100%
G	1023 / 341	NADP-dependent oxidoreductase (*Burkholderia gladioli*) WP_042287319.1	100%
1	819 / 373	IS5 family transposase (*Burkholderia gladioli*) WP_088555423.1	100%

aa[#] : amino acids

Table S2. IC_{50} values of compounds **2-7** toward five cancer cell lines (μM).

Compounds	HCT116	MCF-7	BT-20	HEK-293T	RAW246.
**2**	>20	>20	>20	>40	20.39±2.44
**3**	>20	>20	>20	>40	31.37±1.18
**4**	>20	>20	>20	29.77±3.09	49.24±2.14
**5**	>20	>20	>20	>40	>40
**6**	18.21±1.73	>20	>20	>40	>40
**7**	>20	>20	11.31±0.69	>40	24.82±2.11
Doxorubicin	0.99±0.13	1.06±0.08	0.96±0.95	0.97±0.02	1.00±0.10

Table S3. Strains, mutants and plasmids used in this study.

**Strains**	**Description**	**Source**
Burkholderia gladioli ATCC 10248	Wild type	CGMCC
E. coli GB05Dir/pSC101-BAD-ETgA-tet	derived from *E. coli* GB2005 by integrating the PBAD-ETgA operon (full length recE, recT, redγ and recA under the arabinose-inducible PBAD promoter) at the ybcC locus, direct cloning host	[7]
E. coli GB08Red	derived from *E. coli* GB2005 by integrating the P_{BAD}-gbaA operon (*redγ*, *redβ*, *redα* and *recA* under the arabinose-inducible P_{BAD} promoter) at the *ybcC* locus	[5]
Caldimonas brevitalea DSM 7029Δ*glb-attB*	glidobactin gene cluster was replaced by *attB* fragment	[3]
Burkholderia thailandensis E264Δ*orpC-attB*	*orpC* gene was replaced by attB fragment	[4]
**Plasmids**		
pBBR1-Rha-Redγ-BAS-km	pBBR1 replicon, km^R, recombinase Redγ-BAS under the control of rhamnose promoter	[6]
pFox-genta-P_{tet}-LTX	$genta^R$, PCR templates to amplify gentamicin resistance gene	[11]
pR6K-lox71-genta-lox66-FleQ	R6K replicon, $genta^R$, PCR templates to amplify gentamicin resistance gene	Our lab
pBR322-amp-tetR-tetO-hyg-ccdB	pBR322 replicon, amp^R, *ccdB* gene, PCR templates to amplify ampicillin resistance gene	[12]
pR6K-oriT-TnpA-kan	R6K replicon, suicide plasmid, km^R, PCR templates to amplify *oriT-TnpA* fragment	[12]
pBeloBAC-cm	pBeloBAC11, the vector for direct cloing, cm^R	[7]
pBeloBAC-cm-Chr2C8	cm^R, the *trans*-AT Chr2C8 gene cluster on the plasmid	This study
pBeloBAC-cm-P_{Genta}-Chr2C8	cm^R and $genta^R$, the trans-AT Chr2C8 gene cluster with the constitutive promoter P_{genta} on the plasmid	This study
pBeloBAC-amp-attP-P_{Genta}-Chr2C8	cm^R, $genta^R$, and amp^R, the trans-AT Chr2C8 gene cluster with the constitutive promoter P_{genta} and transposon element *amp-attP* on the plasmid	This study
**Mutants**		
B. gladioli ATCC 10248*ΔgbnΔChr2C8*	The region (2694235-2695549) of Chr2C2 was replaced by gentamicin resistance gene, and the fragment (468577-469853) of gladiolin gene cluster was replaced by apramycin resistance gene in ATCC 10248	This study
B. gladioli ATCC	The P_{Genta} promoter and gentamicin resistance gene	This study

10248*ΔgbnP*$_{Genta}$-*Chr2C8*	was inserted upstream of core biosynthetic region (2702169-2702343) of Chr2C2, and the fragment (468577-469853) of gladiolin gene cluster was replaced by apramycin resistance gene in ATCC 10248	
S. brevitalea DSM 7029Δ*glb*-*Chr2C8*	The Chr2C8 gene cluster inserted into glidobactin gene by attP/B site in the heterologous host *S. brevitalea* DSM 7029Δ*glb*	This study
B. thailandensis E264-*Chr2C8*	The Chr2C8 gene cluster inserted into genome by attP/B site in the heterologous host *B. thailandensis* E264	This study

Table S4. Primers used in this study.

Primers	Primer sequences (5'-3')
Chr2C8-10248-Pgenta-S	TCGGAGCCGAAAGAGGCTTGTCCATGTCTTGCCAGATAAAGACTTTGCATAATCTGTACCTCCTTAAGTGA
Chr2C8-10248-Pgenta-A	GTCCTTGCTTGGCTTGGCTTATCGTGCGTCGCAGTATGACTGTGGTGCTGGAAGGCACGAACCCAGTTGA
Chr2C8KO-10248-genta-S	GACCTGGAGCGCGTCGGCCAGGTTGGCTGCATCGGCCCCGGCGAGGCTCGAATCTGTACCTCCTTAAGTGA
Chr2C8KO-10248-genta-A	CAGCCGAGCATGCCACCGCCGCGCAGCCGCTCGCCGAAGCGGCCTCGGCAGAAGGCACGAACCCAGTTGA
pBeloBAC-cm-10248Chr2C8-S	CGAACCTCGCCAGCATGCCCTGCACAAAAATCCACAGGAAGCCCGGCCGGCCGGGCTTGACGCGGCTTCGCCGGGCTCCACGCTGATAGTCTGATCGACA
pBeloBAC-cm-10248Chr2C8-A	GCCCGAAACACGGTGCTCTGGCCGGCTGGCAGGCGTTCCAGCCTGCCTCGCGCGAGGCAGGCTGGATAGGCCAAACATAGGGCACCAATAACTGCCTTAA
BAC-BGC8-amp-attP-S	GGTTCCAACTTTCACCATAATGAAATAAGATCACTACCGGTTTGTTTATTTTTCTAAATACATTC
BAC-BGC8-amp-attP-A	AGGCAGGCTGGATAGGCCAAACATAGGGCACCAATAACTGCTGAGCGGAGAACGAGATGAC
Chr2C8Pgenta-check-S	TTCATCGAAGCCGGCATCGA
Chr2C8Pgenta-check-A	ATCGATTCGTCCCTGCCATG
Chr2C8KO-check-S	ATCTGCAGCGTGTAGGCGAG
Chr2C8KO-check-A	TACGTAAGGCGCGAGGGTCT
Check1-primer-S	TATGATCTCGCAGTCTCCGG
Check1-primer-A	GCATGAACAACGCGGGAT
Check2-primer-S	GATGGCGCTCGTCTTCAAAT
Check2-primer-A	CTCGCCGTTGAAACAGATCC

References

1. Blin, K; Shaw, S; Augustijn, H. E.; Reitz, Z. L; Biermann, F.; Alanjary, M.; Fetter, A.; Terlouw, B. R.; Metcalf, W. W.; Helfrich, E. J. N.; van Wezel, G.P.; Medema, M. H.; Weber, T. antiSMASH 7.0: new and improved predictions for detection, regulation, chemical structures and visualisation. *Nucleic Acids Res*., **2023**, *51*, W46-W50.
2. Mistry, J.; Chuguransky, S.; Williams, L.; Qureshi, M.; Salazar, G. A.; Sonnhammer, E. L. L.; Tosatto, S. C. E.; Paladin, L.; Raj, S.; Richardson, L. J.; Finn, R. D.; Bateman, A. Pfam: The protein families database in 2021. *Nucleic Acids Res*. **2021**, *49*, D412-D419.
3. Liu, J.Q.; Zhou, H.B.; Yang, Z.Y.; Wang, X.; Chen, H.N.; Zhong, L.; Zheng, W.T.; Niu, W.J.; Wang, S.; Ren, X.M.; Zhong, G.N.; Wang, Y.; Ding, X.M.; Müller, R.; Zhang, Y.M.; Bian, X.Y. Rational construction of genome-reduced Burkholderiales chassis facilitates efficient heterologous production of natural products from proteobacteria. *Nat. Commun*., **2021**, *12*, 4347.
4. Wang, Z.J.; Liu, X.T.; Zhou, H.B.; Liu, Y.; Zhong, L.; Wang, X.; Tu, Q.; Huo, L.J.; Yan, F.; Gu, L.C.; Müller, R.; Zhang, Y.M.; Bian, X.Y.; Xu, X.K. Engineering of *Burkholderia thailandensis* strain E264 serves as a chassis for expression of complex specialized metabolites. *Front. Microbiol.*, **2022**, *13*, 1073243.
5. Fu J.; Teucher M.; Anastassiadis K.; Skarnes W.; Stewart A. F. A recombineering pipeline to make conditional targeting constructs. *Methods Enzymol*., **2010**, *477*, 125–144.
6. Chen, H.N.; Sun, T.; Bai, X.P.; Yang, J.; Yan, F.; Yu, L.; Tu, Q.; Li, A.Y.; Zhang, Y.M.; Bian, X.Y.; Zhou, H.B. Genomics-driven activation of silent biosynthetic gene clusters in *Burkholderia gladioli* by screening recombineering system. *Molecules*, **2021**, *26*, 700.
7. Wang, H.L.; Li,Z.; Jia, R.N.; Yin, J.; Li, A.Y.; Xia, L.Q.; Yin, Y.L.; Müller, R.; Fu, J.; Stewart, A.F.; Zhang, Y. ExoCET: exonuclease in vitro assembly combined with RecET recombination for highly efficient direct DNA cloning from complex genomes. *Nucleic Acids Res.*, **2018**, *46*, e28.
8. Bonis, B.M.; Gralnick, J.A. Marinobacter subterrani, a genetically tractable neutrophilic Fe (II)-oxidizing strain isolated from the Soudan Iron Mine. *Front. Microbiol.*, **2015**, *6*, 719.
9. Wang, X.; Zhou, H.B.; Chen, H.N.; Jing, X.S.; Zheng, W.T.; Li, R.J.; Sun, T.; Liu, J.Q.; Fu, J.; Huo, L.J.; Li, Y.Z.; Shen, Y.M.; Ding, X.M.; Müller, R.; Bian, X.Y.; Zhang, Y.M. Discovery of recombinases enables genome mining of cryptic biosynthetic gene clusters in Burkholderiales species. *Proc. Natl. Acad. Sci. U S A*., **2018**, *115*, E4255-E4263.
10. Li, R.J.; Shi, H.B.; Zhao, X.Y.; Liu, X.Q.; Duan, Q.; Song, C.Y.; Chen, H.N.; Zheng, W.T.; Shen, Q.Y.; Wang, M.Q.; Wang, X.; Gong, K.; Yin, J.; Zhang, Y.M.; Li, A.Y.; Fu, J. Development and application of an efficient recombineering system for *Burkholderia glumae* and *Burkholderia plantarii*. *Microb. Biotechnol*., **2021**, *14*, 1809-1826.
11. Ongley, S.E.; Bian, X.Y.; Zhang, Y.M.; Chau, R.; Gerwick, W.H.; Müller, R.; Neilan, B.A. High-titer heterologous production in E. coli of lyngbyatoxin, a protein kinase C activator from an uncultured marine cyanobacterium. *ACS Chem. Biol*., **2013**, *8*, 1888-1893.
12. Wang, H.L.; Li, Z.; Jia, R.N.; Hou, Y.; Yin, J.; Bian, X.Y.; Li, A.Y.; Müller, R; Stewart, A.F.; Fu, J.; Zhang, Y.M. RecET direct cloning and Red$\alpha\beta$ recombineering of biosynthetic gene clusters, large operons or single genes for heterologous expression. *Nat. Protoc.*, **2016**, *11*, 1175–1190.

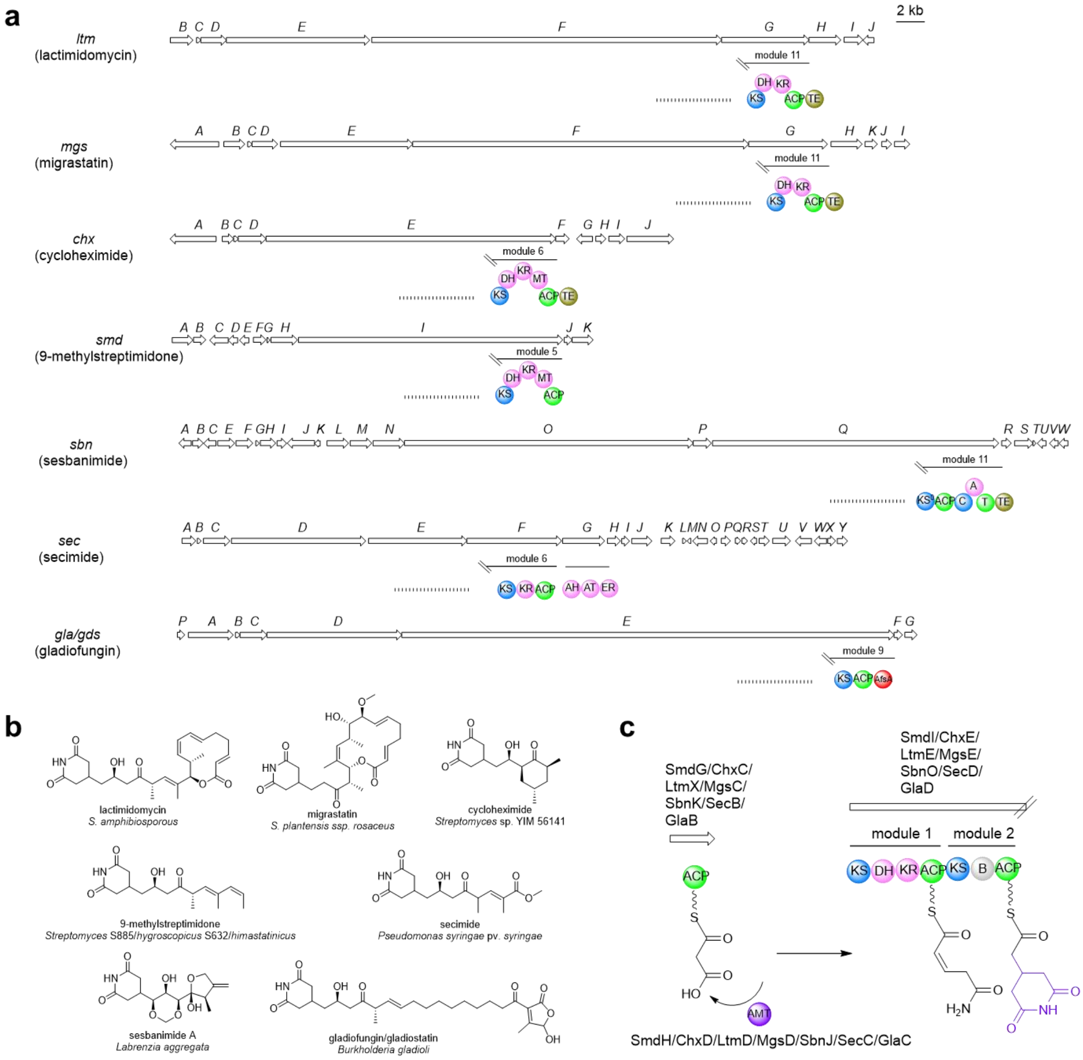

Figure S1. Comparison of biosynthetic gene clusters (BGCs) encoding glutarimide-containing antibiotics in different strains. (a) Diagram of glutarimide BGCs and C-terminal domains. (b) The chemical structures of glutarimide-containing antibiotics produced by different strains. (c) The formation of glutarimide moiety that is encoded by corresponding enzymes.

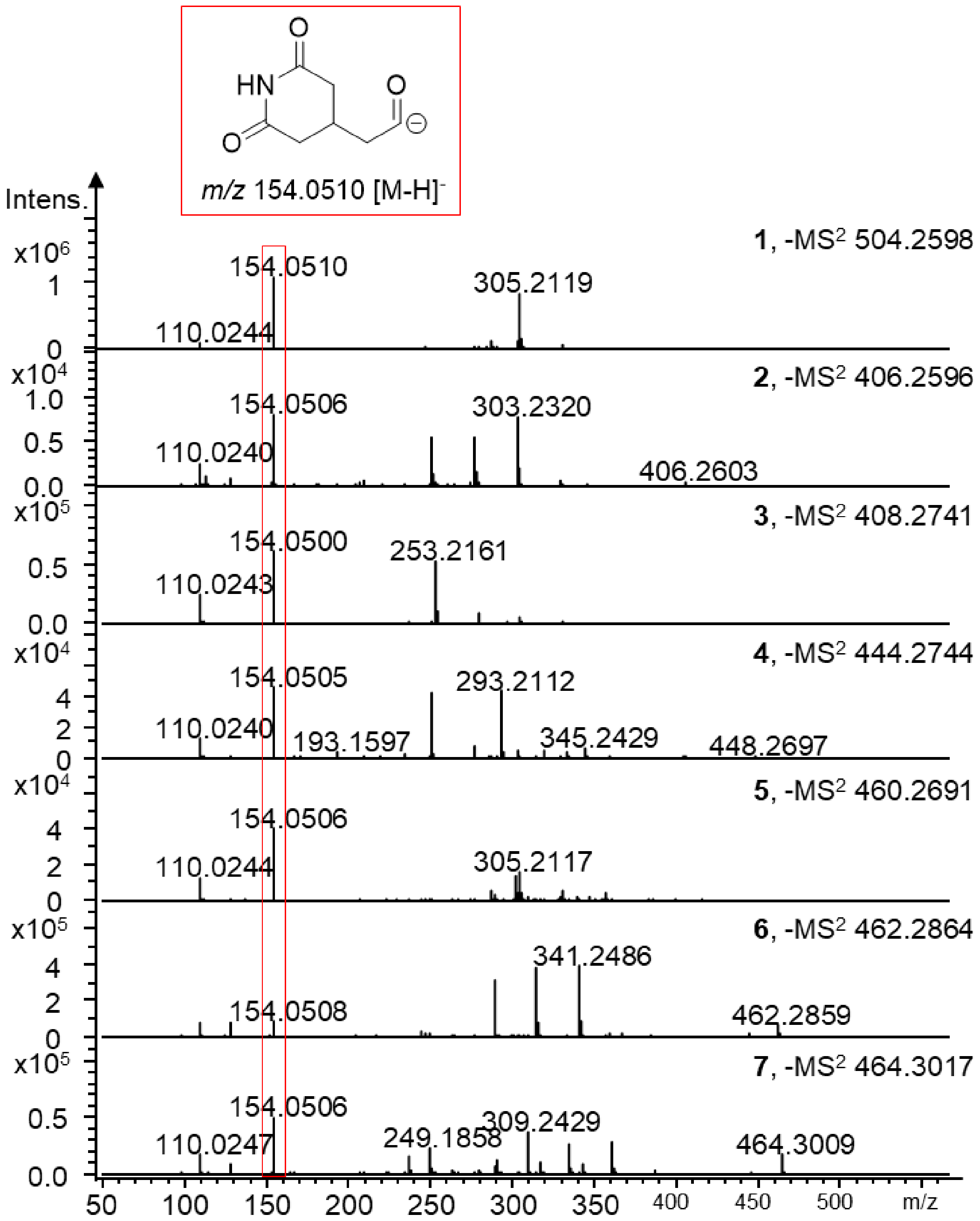

Figure S2. HPLC-MS/MS analysis of compounds **1**-**7**.

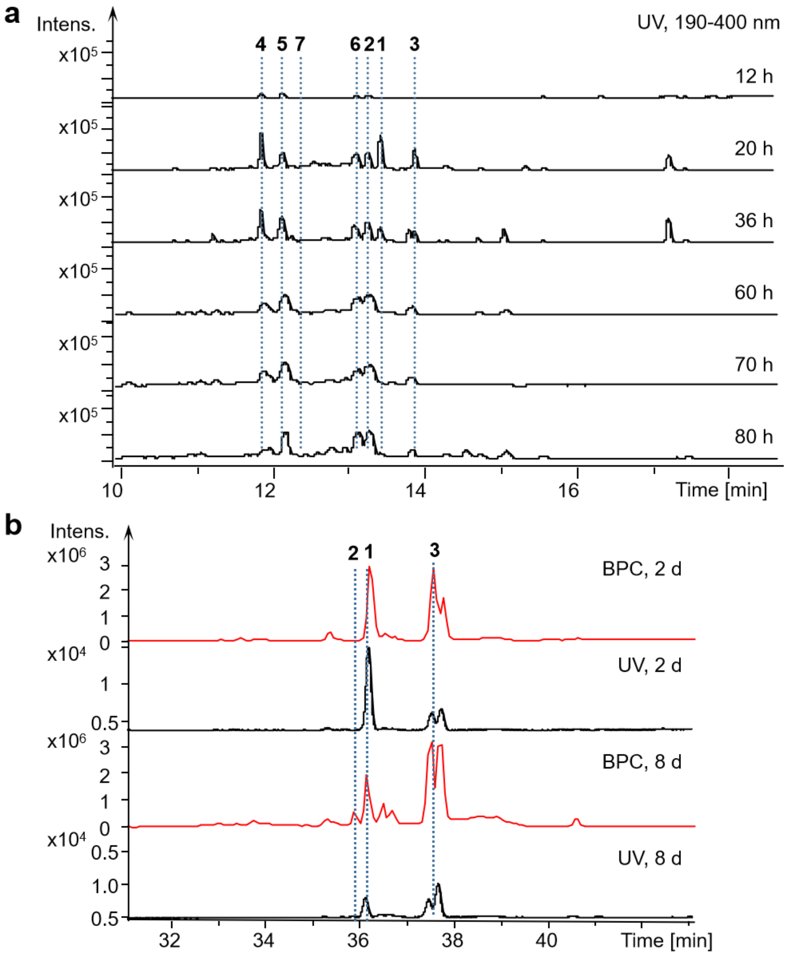

Figure S3. LC-MS analysis of crude extracts and compound **1** at different time intervals. (a) LC-MS analysis of crude extracts from mutant ATCC 10248Δ*gbn*:P_{Genta}-Chr2C8 at different time intervals using ethyl acetate at room temperature. (b) HPLC-MS analysis of purified **1** (glidofungin A) stored in methanol solvent for 2 days and 8 days after purification at room temperature. BPC: 100-1500, UV:190-400 nm.

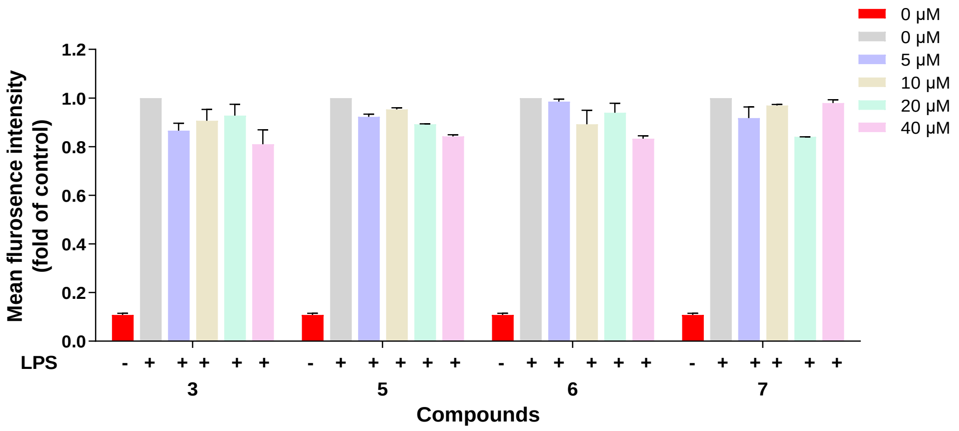

Figure S4. Inhibition of compounds **3**, **5**, **6**, and **7** on LPS-induced NO production in RAW 264.7 macrophages. The concentrations of compounds were 5 μM, 10 μM, 20 μM, and 40 μM.

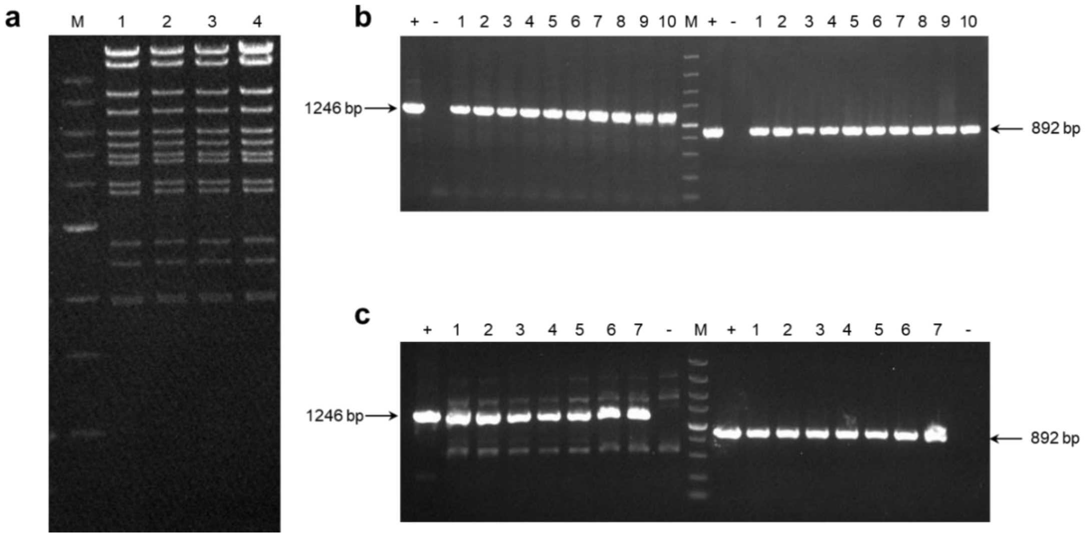

Figure S5. Verification of the cloning and engineering of *gla* gene cluster. (a) Digestion of final plasmid p15A-attP-amp-P_{Genta}-*gla* by enzyme PvuII, M: 1 kb DNA ladder, (b) Colony PCR verification of transformants of *Schlegella brevitalea* DSM 7029, (c) Colony PCR verification of transformants of *Burkholderia thailandensis* E264. M: DL5000 DNA ladder. +: positive control, -: negative control.

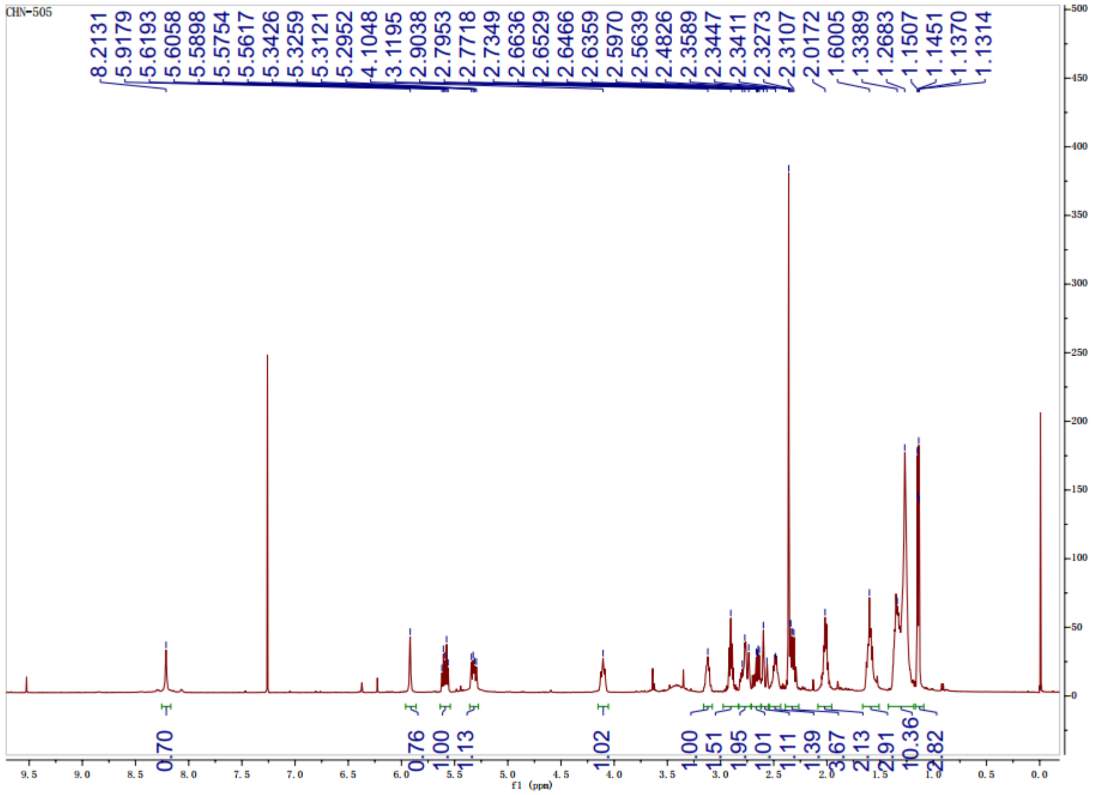

Figure S6. ^{1}H NMR spectrum of gladiofungin A (**1**) in $CDCl_3$

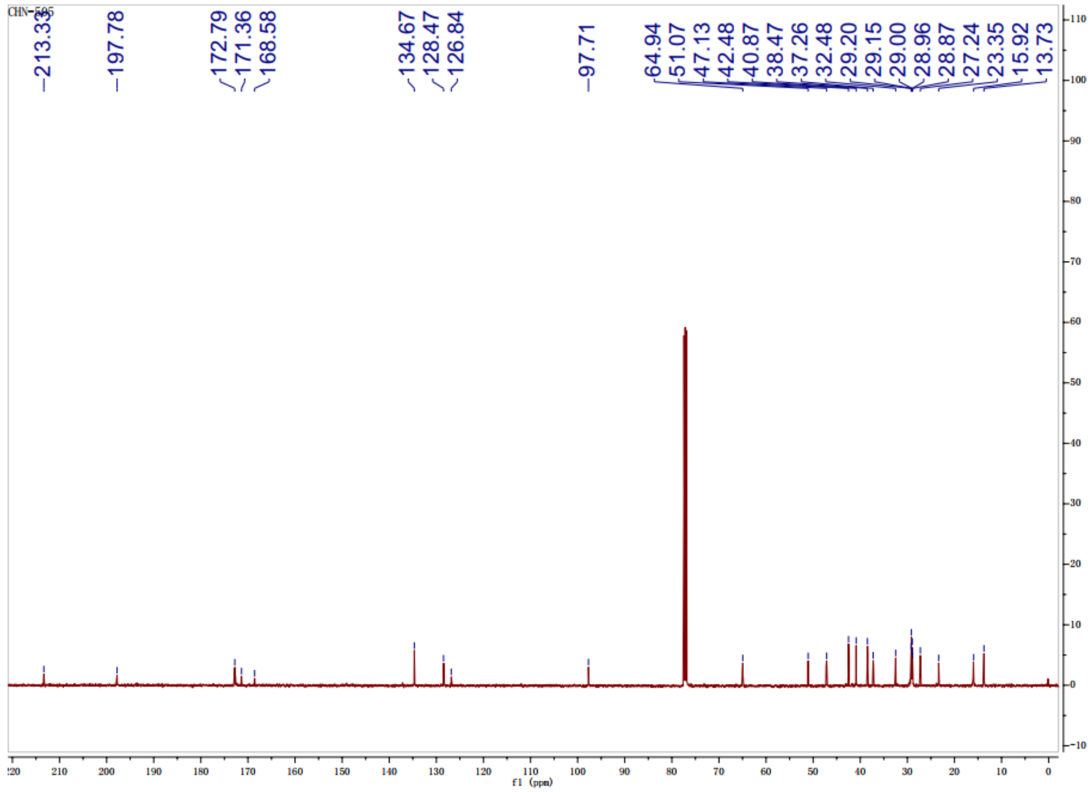

Figure S7. ^{13}C NMR spectrum of gladiofungin A (**1**) in $CDCl_3$

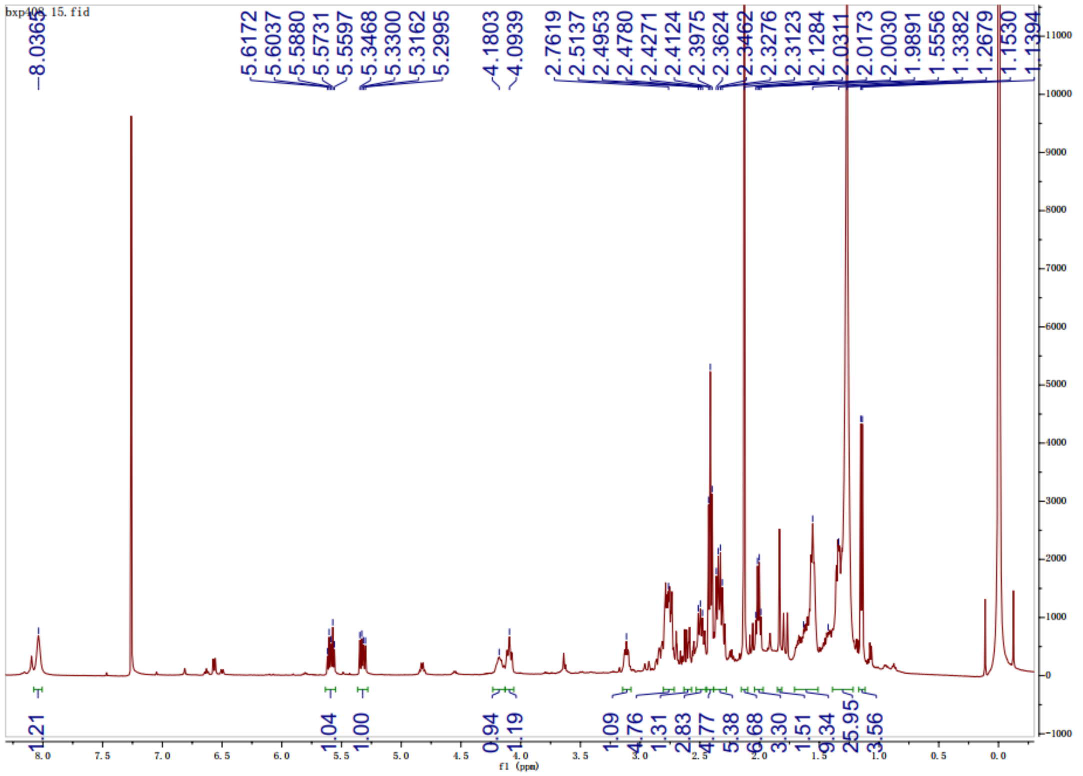

Figure S8. ^{1}H NMR spectrum of gladiofungin C (**2**) in $CDCl_3$

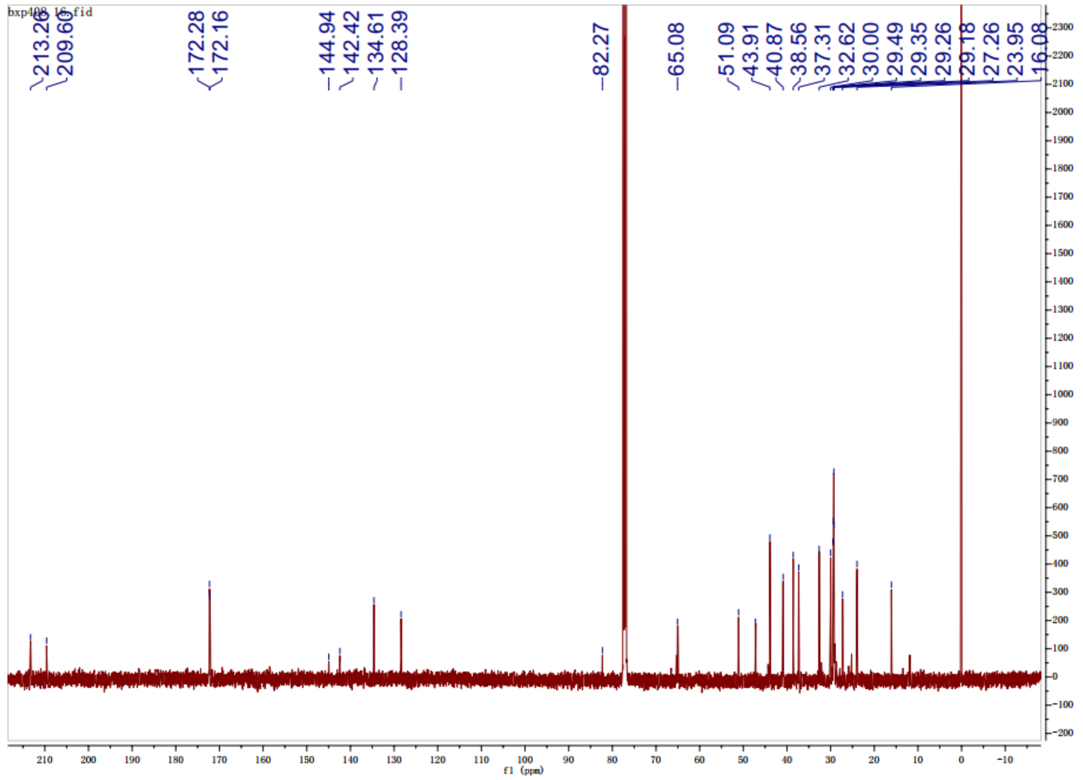

Figure S9. ^{13}C NMR spectrum of gladiofungin C (**2**) in $CDCl_3$

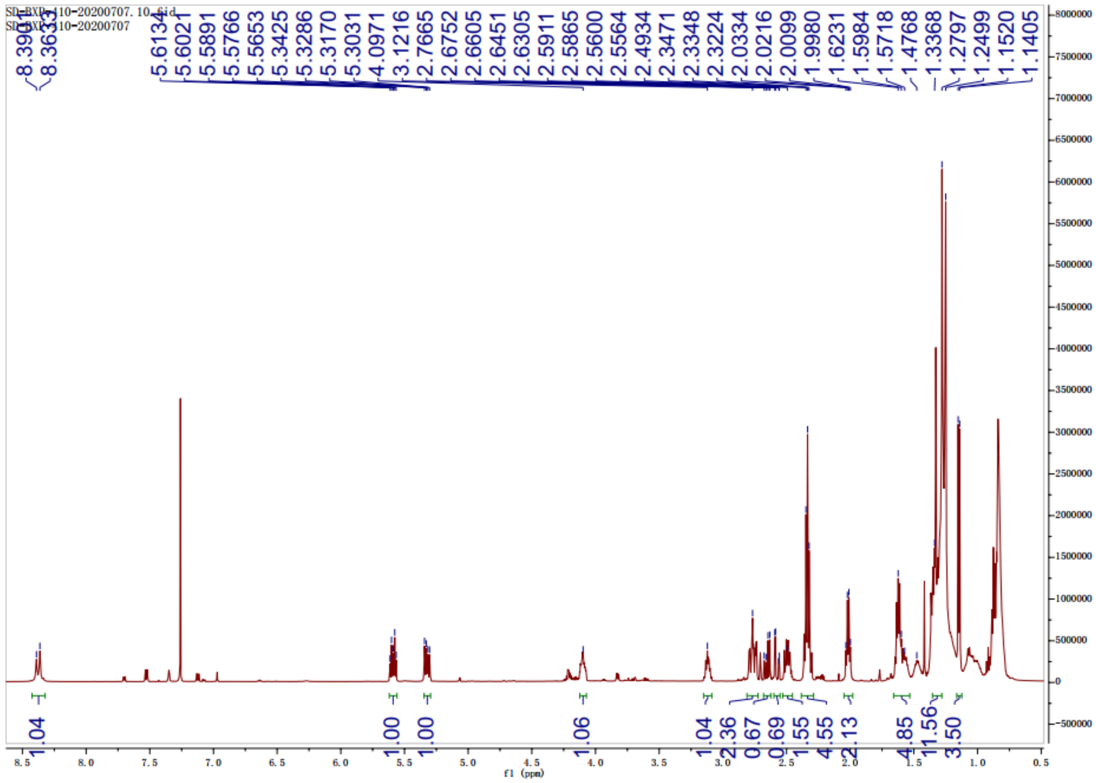

Figure S10. ^{1}H NMR spectrum of gladiofungin D (**3**) in $CDCl_3$

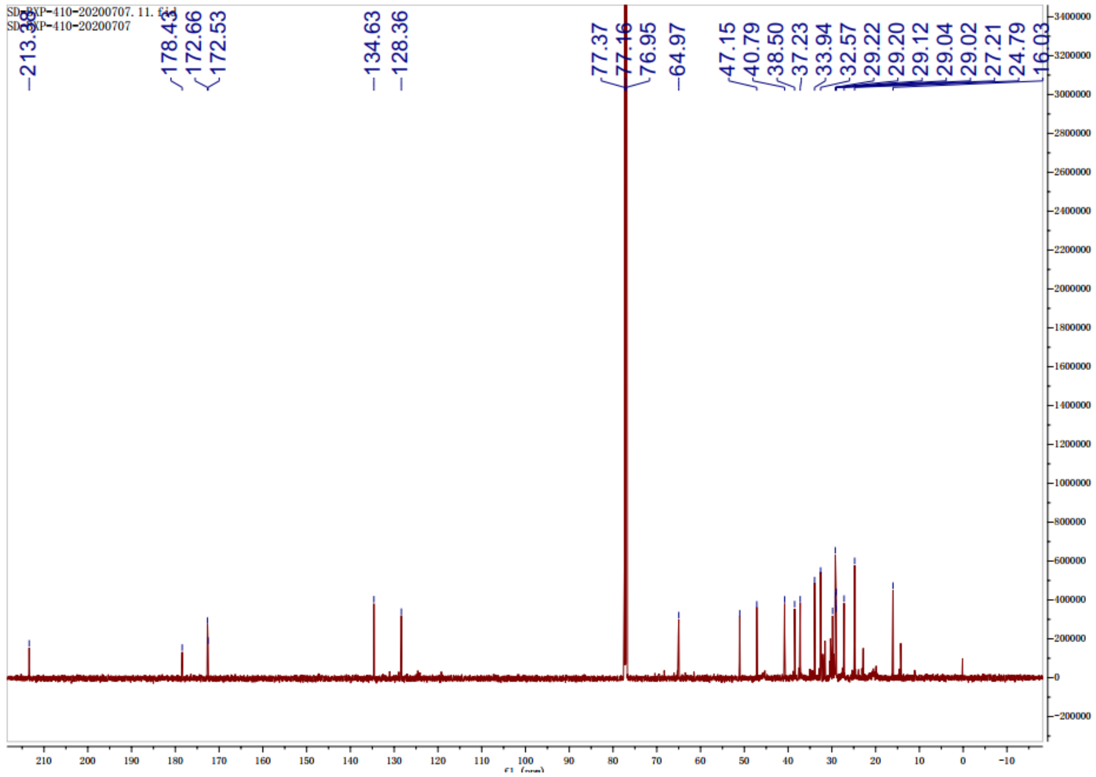

Figure S11. ^{13}C NMR spectrum of gladiofungin D (**3**) in $CDCl_3$

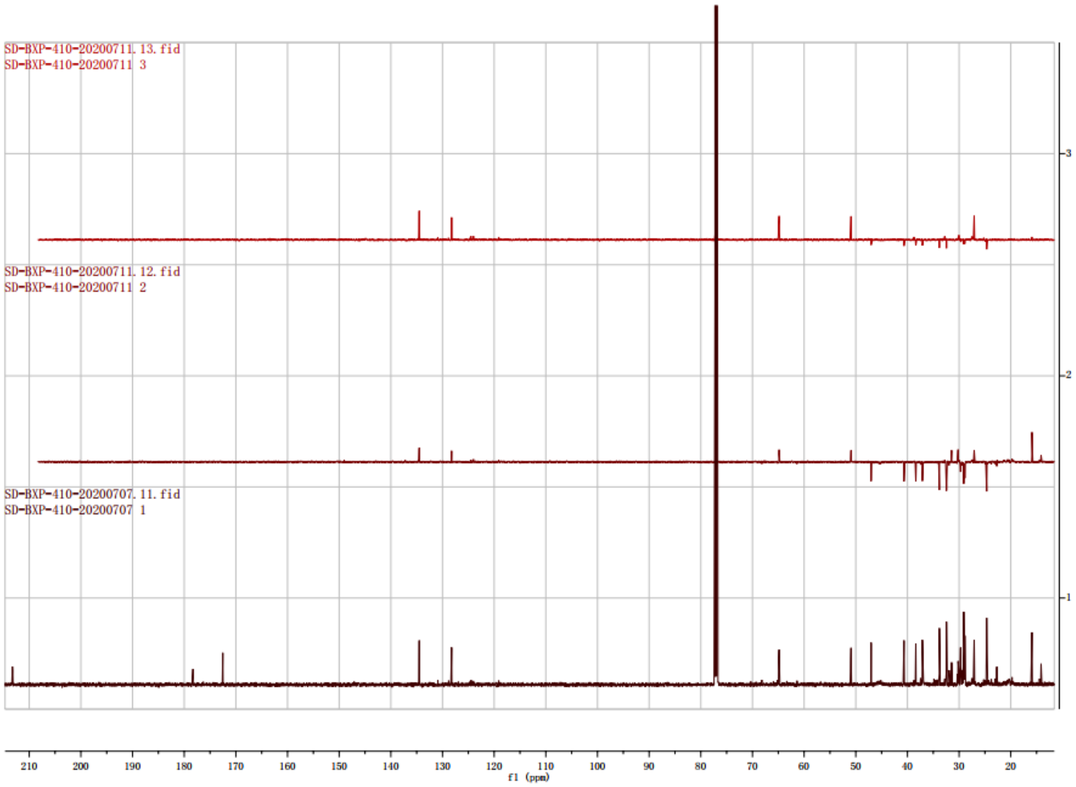

Figure S12. DEPT spectrum of gladiofungin D (**3**) in $CDCl_3$

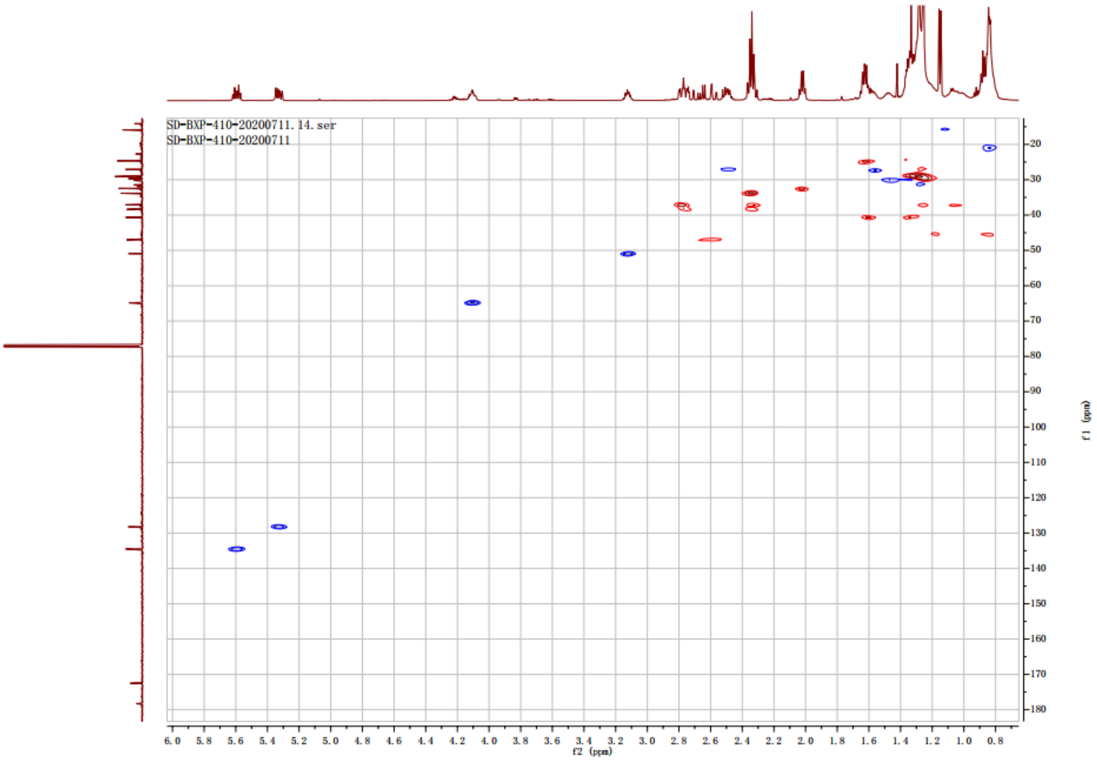

Figure S13. HSQC spectrum of gladiofungin D (**3**) in $CDCl_3$

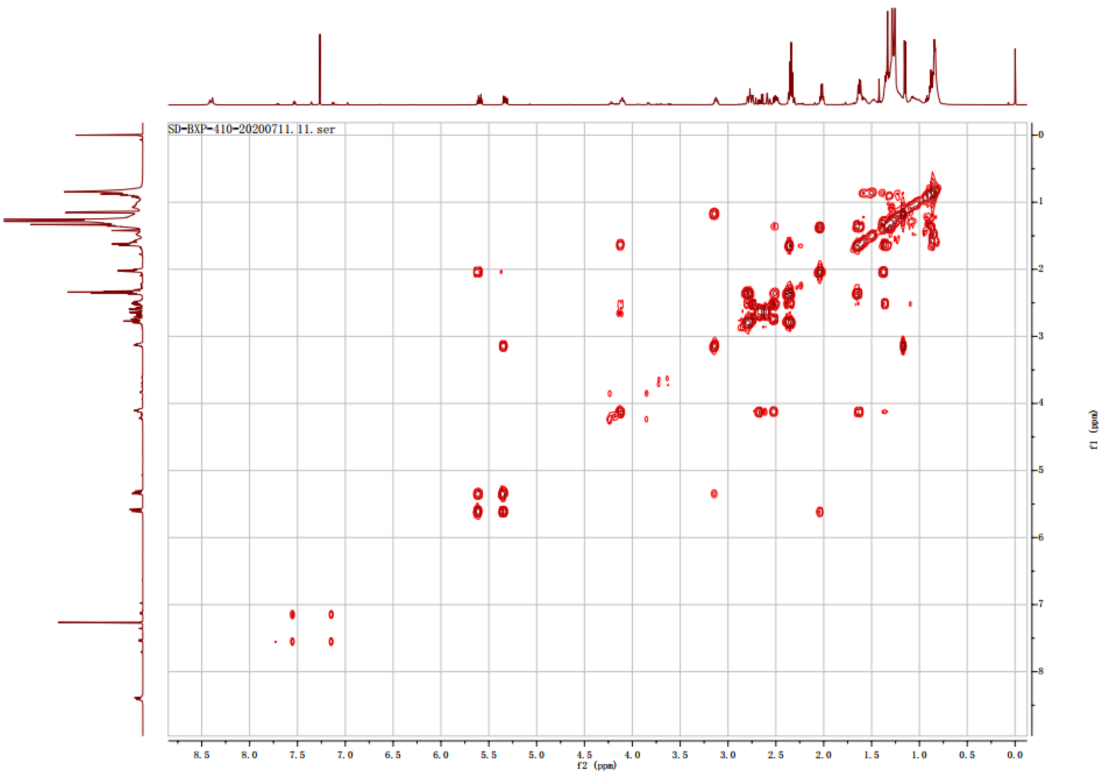

Figure S14. ^{1}H-^{1}H COSY spectrum of gladiofungin D (**3**) in $CDCl_3$

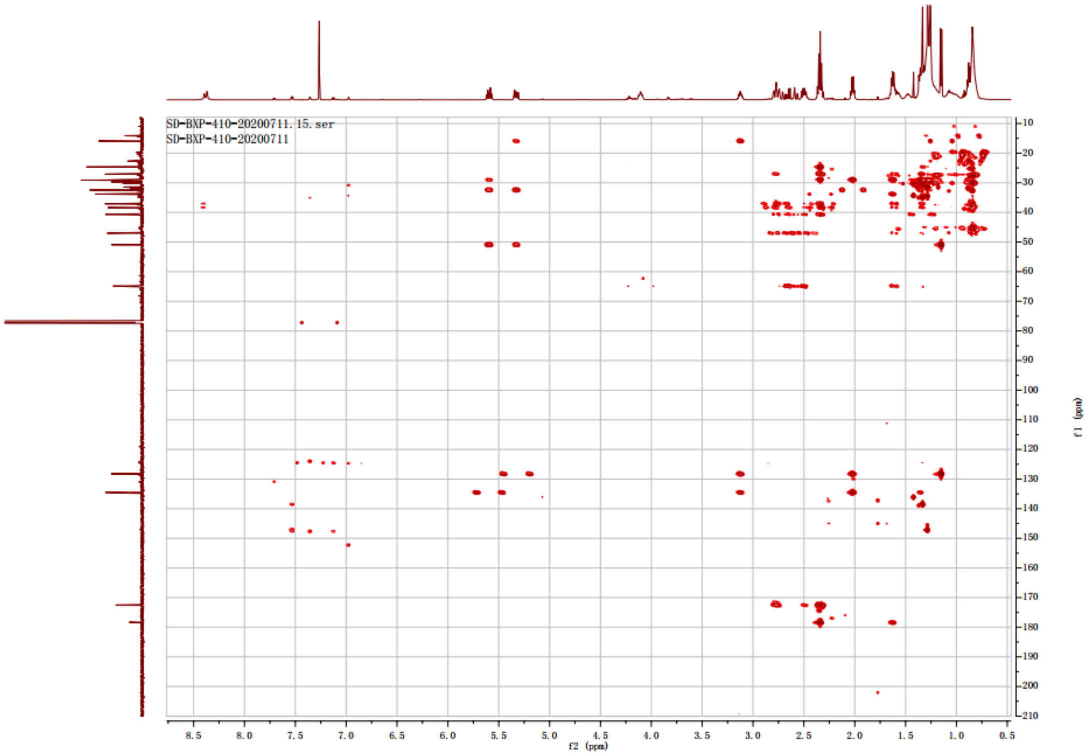

Figure S15. HMBC spectrum of gladiofungin D (**3**) in $CDCl_3$

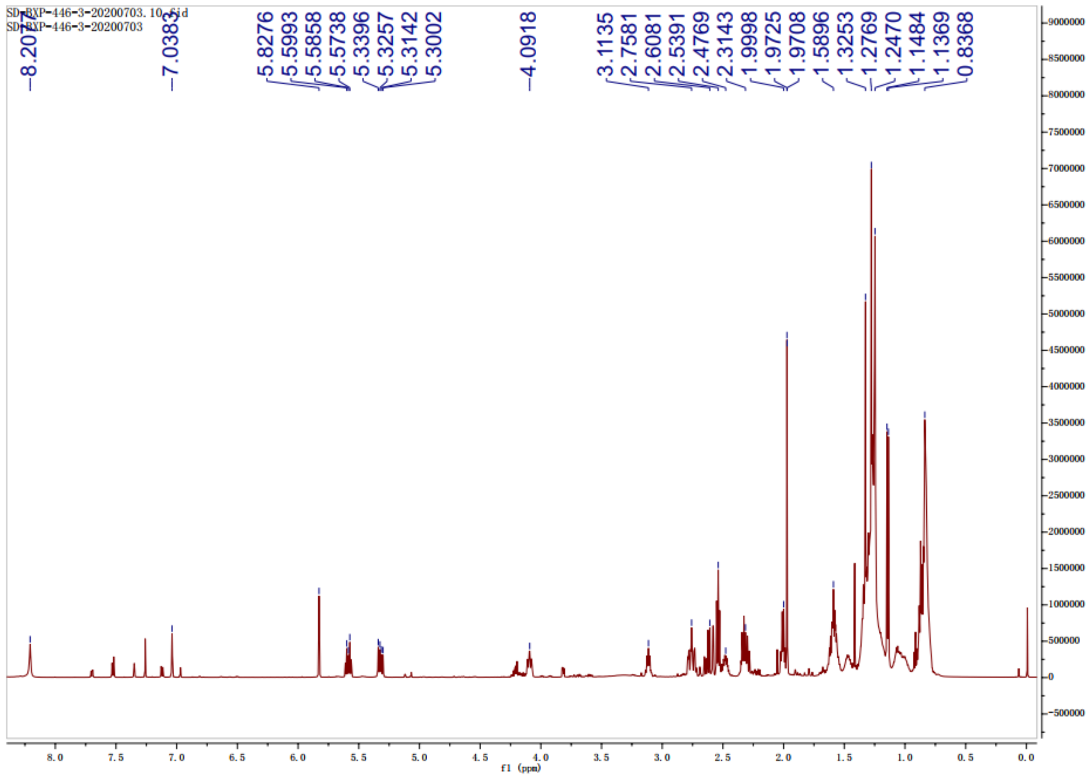

Figure S16. ^{1}H NMR spectrum of gladiofungin E (**4**) in $CDCl_3$

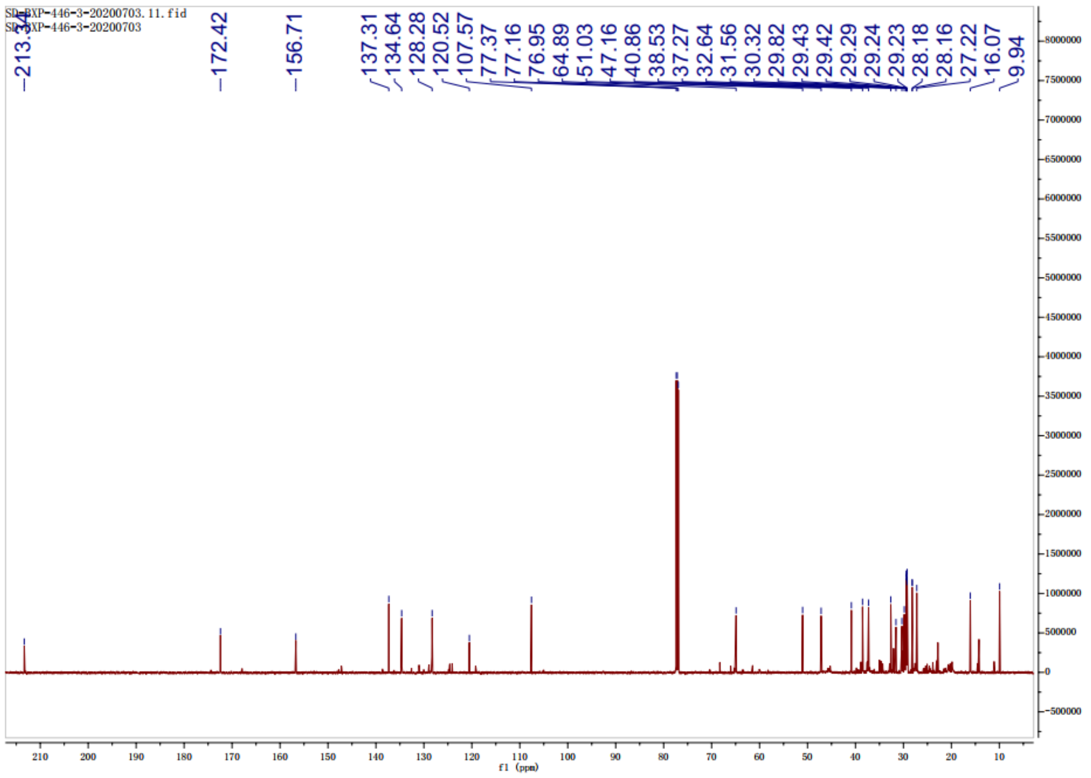

Figure S17. ^{13}C NMR spectrum of gladiofungin E (**4**) in $CDCl_3$

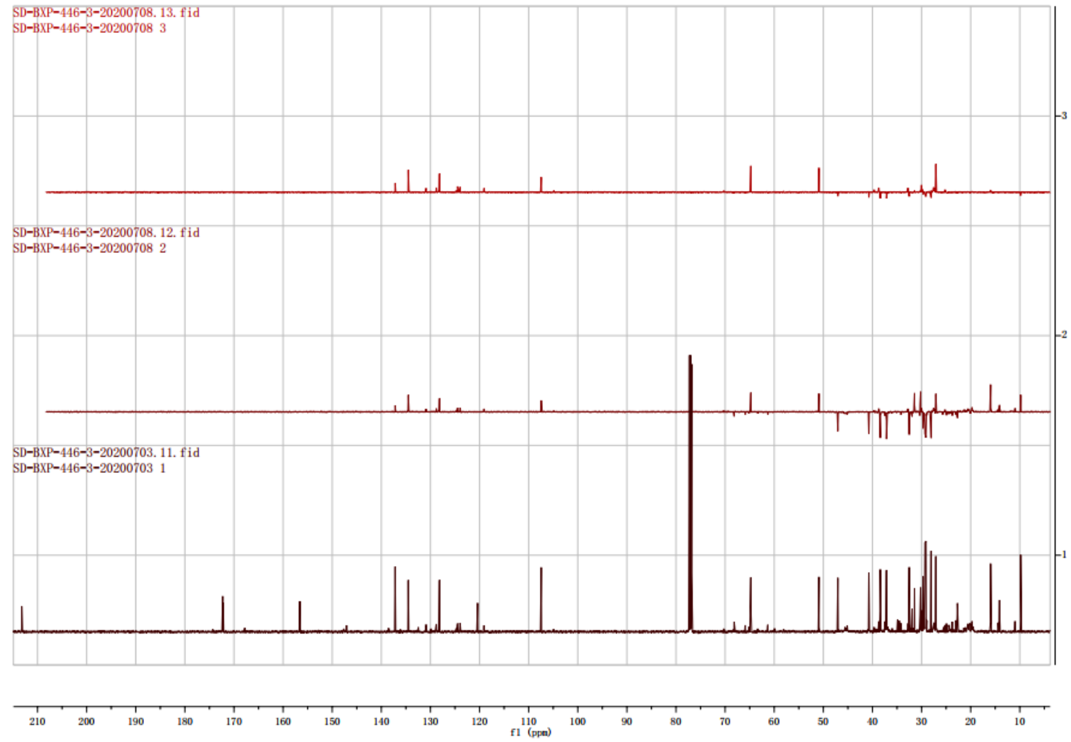

Figure S18. DEPT spectrum of gladiofungin E (**4**) in $CDCl_3$

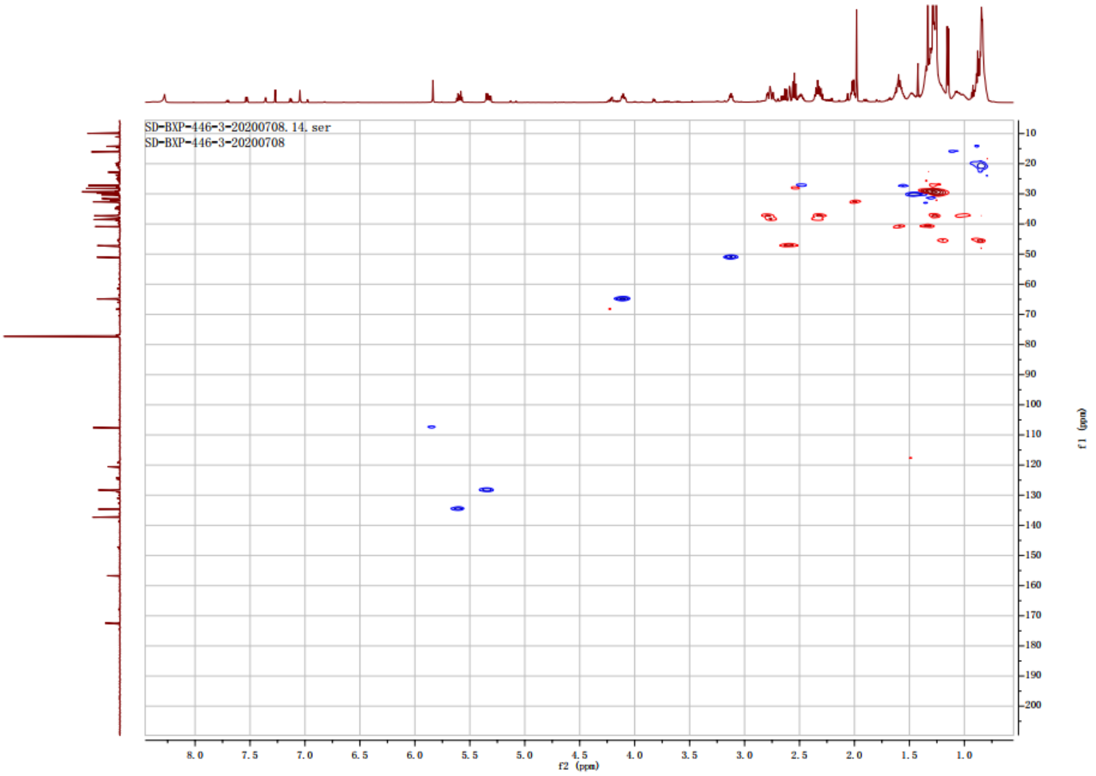

Figure S19. HSQC spectrum of gladiofungin E (**4**) in $CDCl_3$

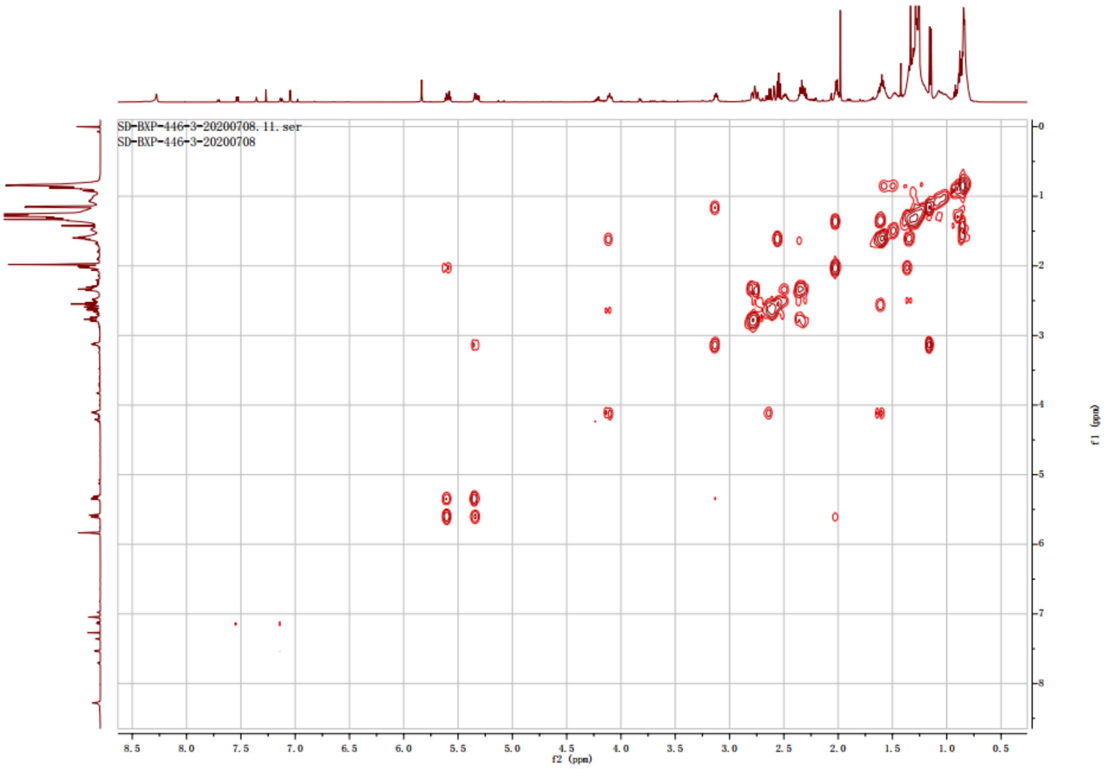

Figure S20. ^{1}H-^{1}H COSY spectrum of gladiofungin E (**4**) in $CDCl_3$

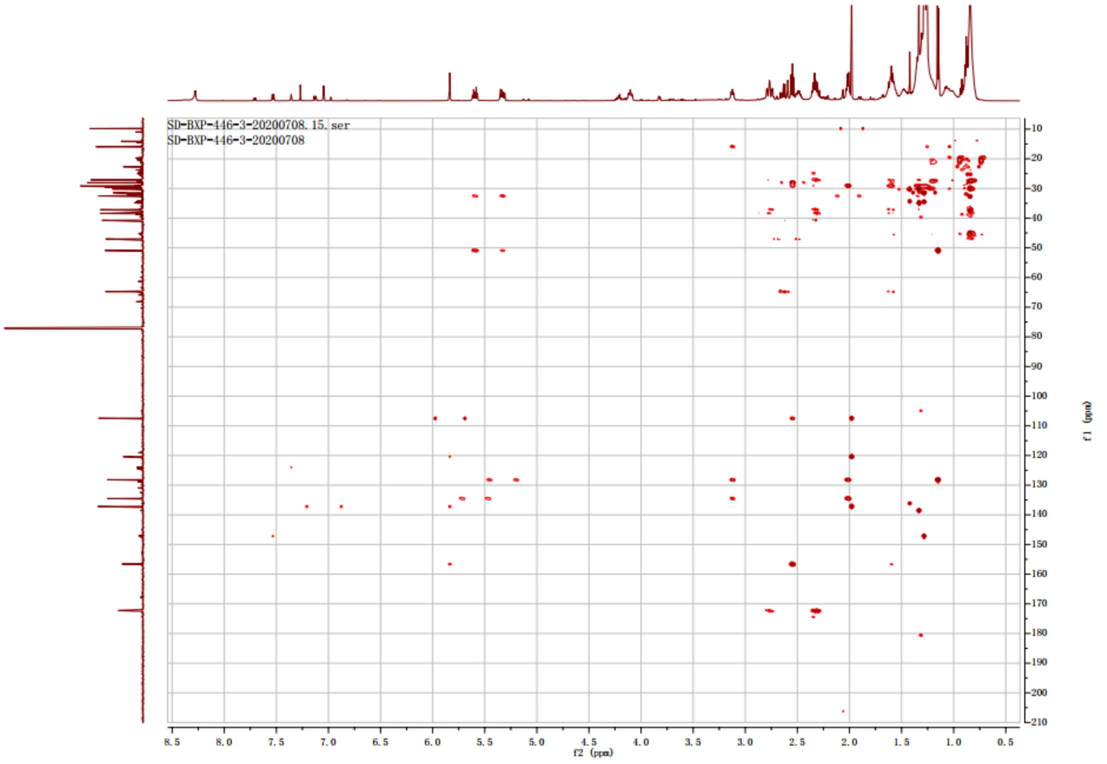

Figure S21. HMBC spectrum of gladiofungin E (**4**) in $CDCl_3$

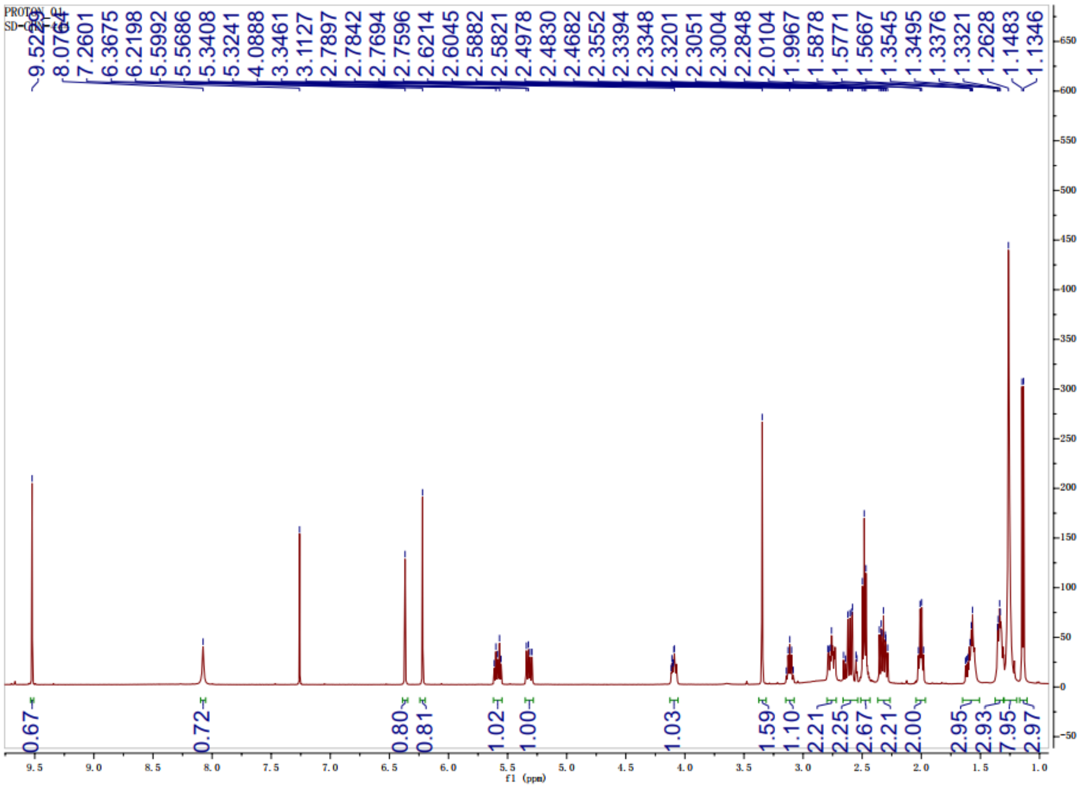

Figure S22. ^{1}H NMR spectrum of gladiofungin F (**5**) in $CDCl_3$

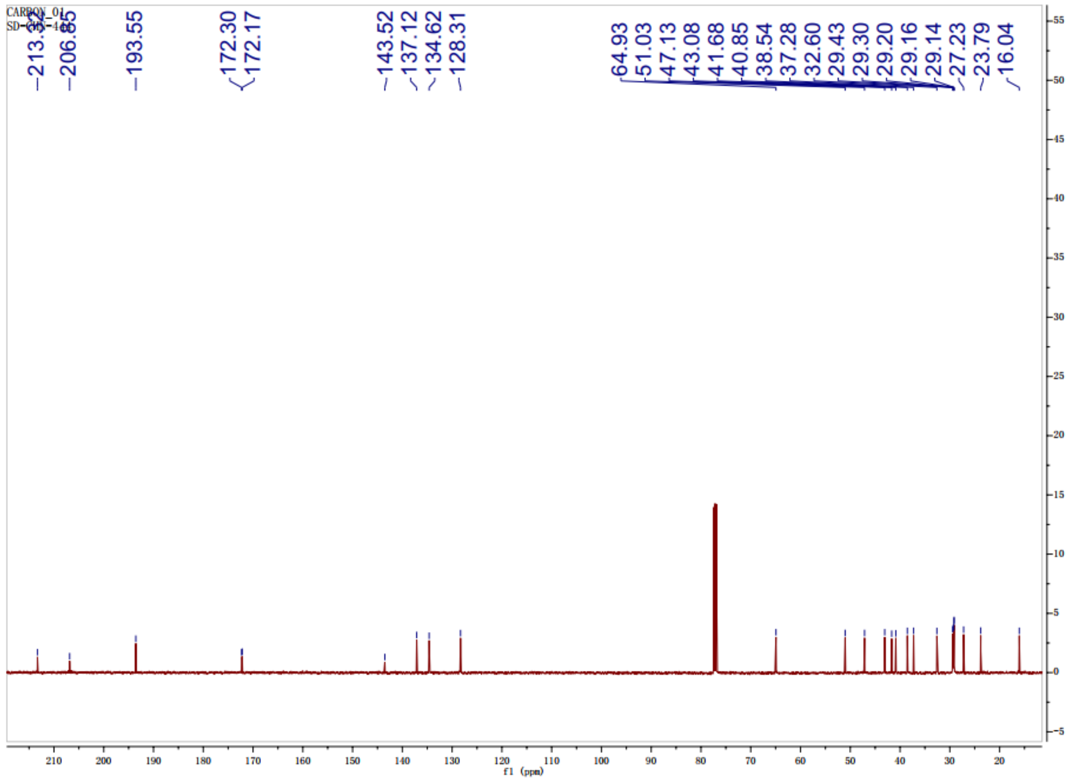

Figure S23. ^{13}C NMR spectrum of gladiofungin F (**5**) in $CDCl_3$

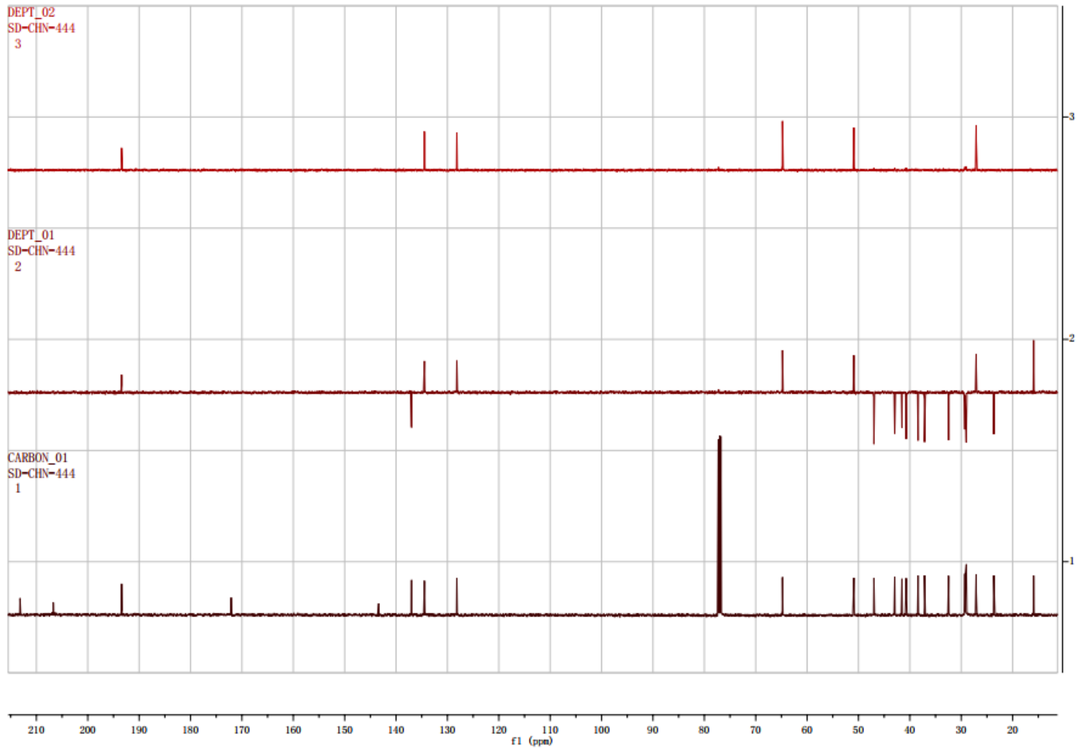

Figure S24. DEPT spectrum of gladiofungin F (**5**) in $CDCl_3$

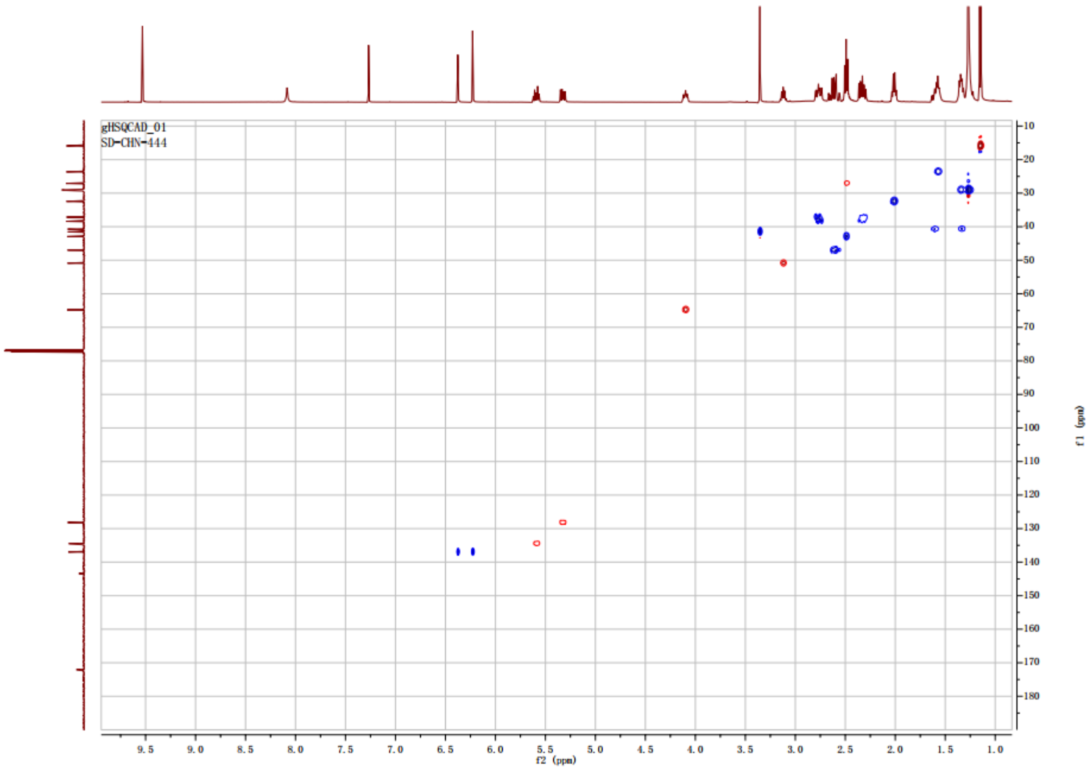

Figure S25. HSQC spectrum of gladiofungin F (**5**) in $CDCl_3$

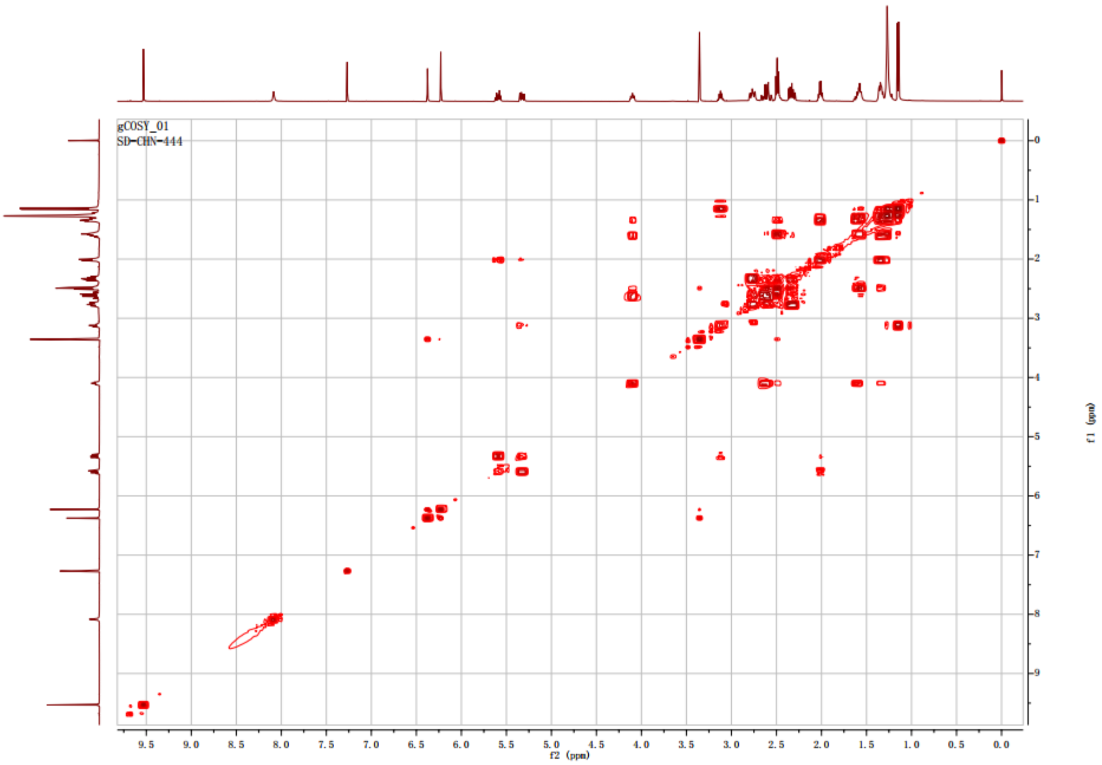

Figure S26. ^{1}H-^{1}H COSY spectrum of gladiofungin F (**5**) in $CDCl_3$

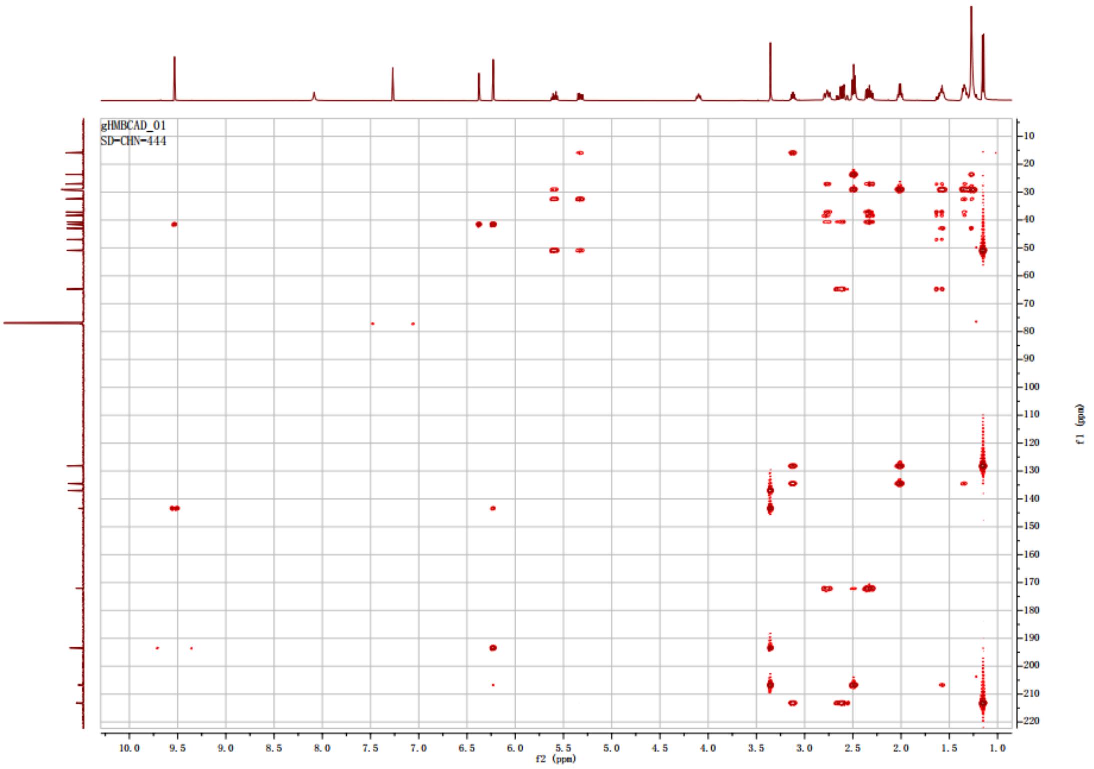

Figure S27. HMBC spectrum of gladiofungin F (**5**) in $CDCl_3$

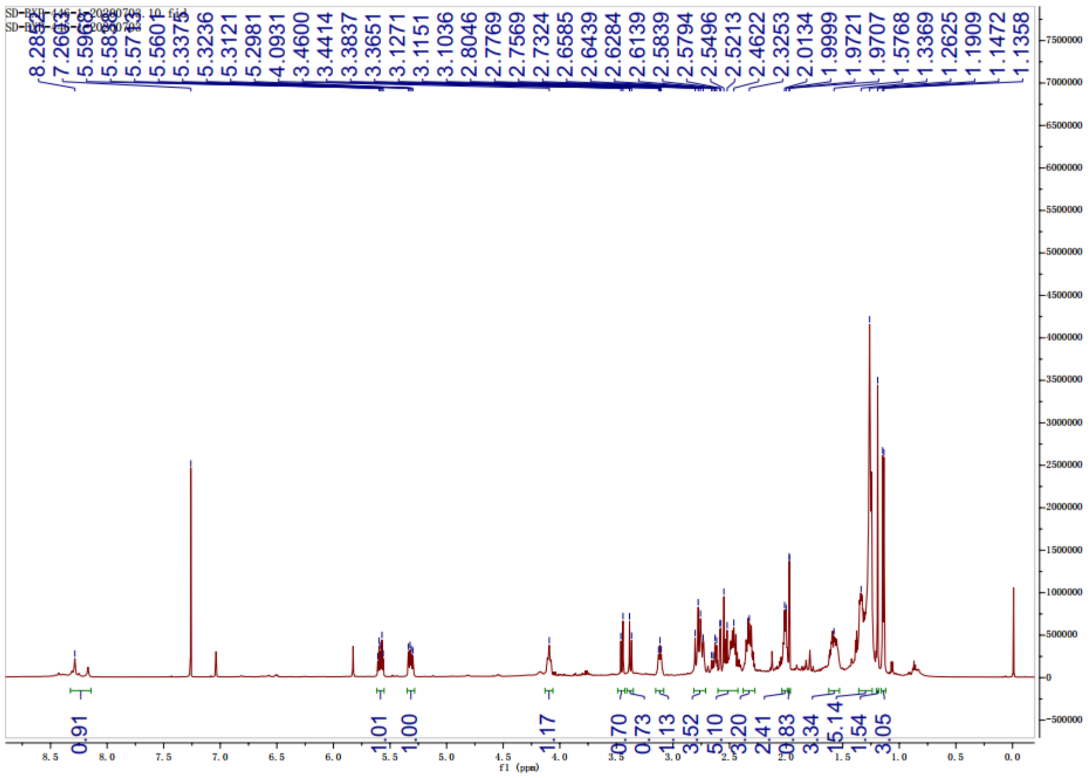

Figure S28. ^{1}H NMR spectrum of gladiofungin G (**6**) in $CDCl_3$

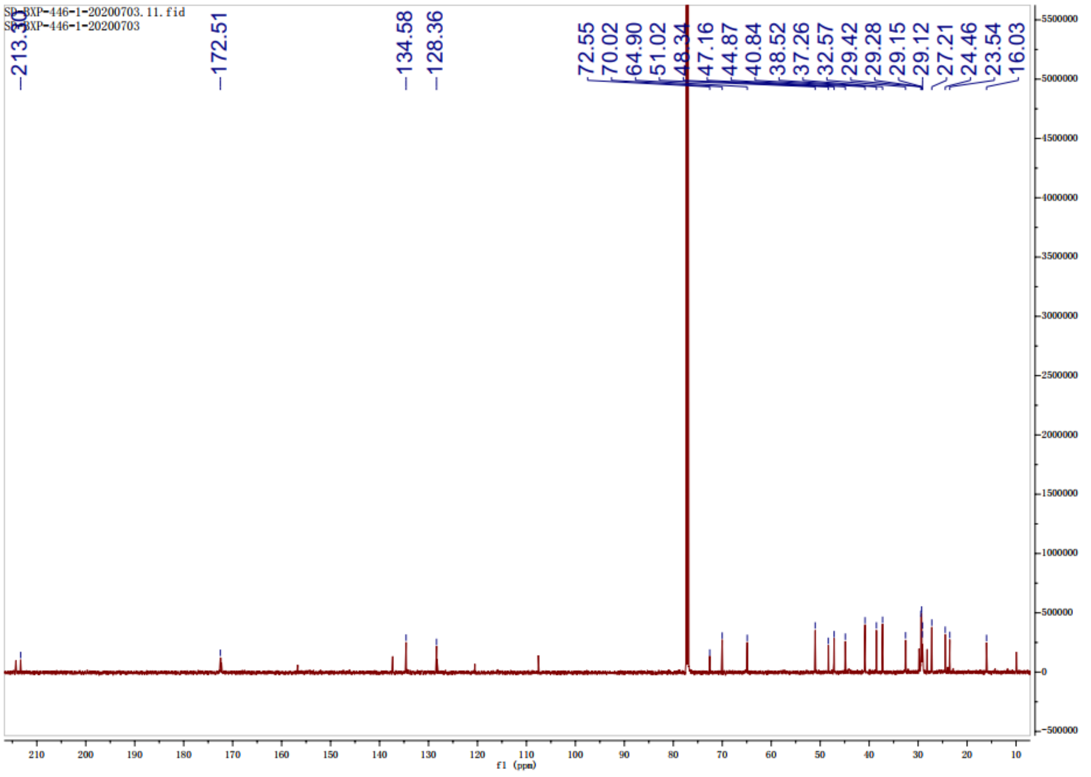

Figure S29. ^{13}C NMR spectrum of gladiofungin G (**6**) in $CDCl_3$

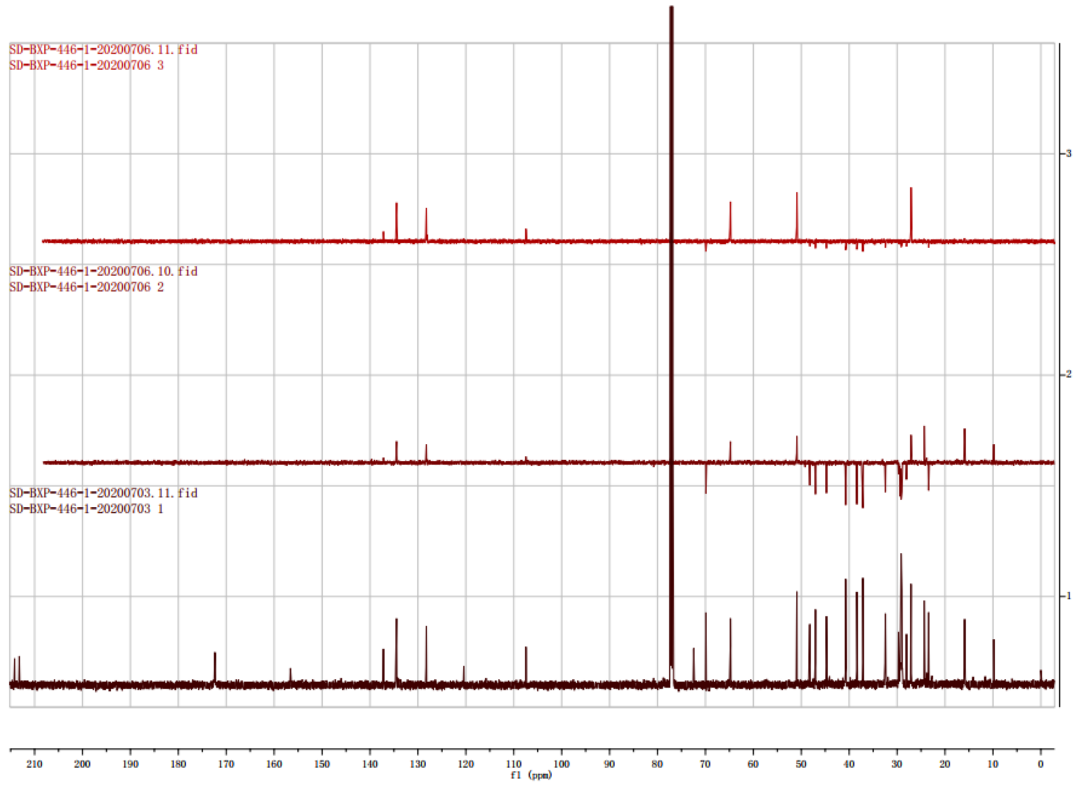

Figure S30. DEPT spectrum of gladiofungin G (**6**) in $CDCl_3$

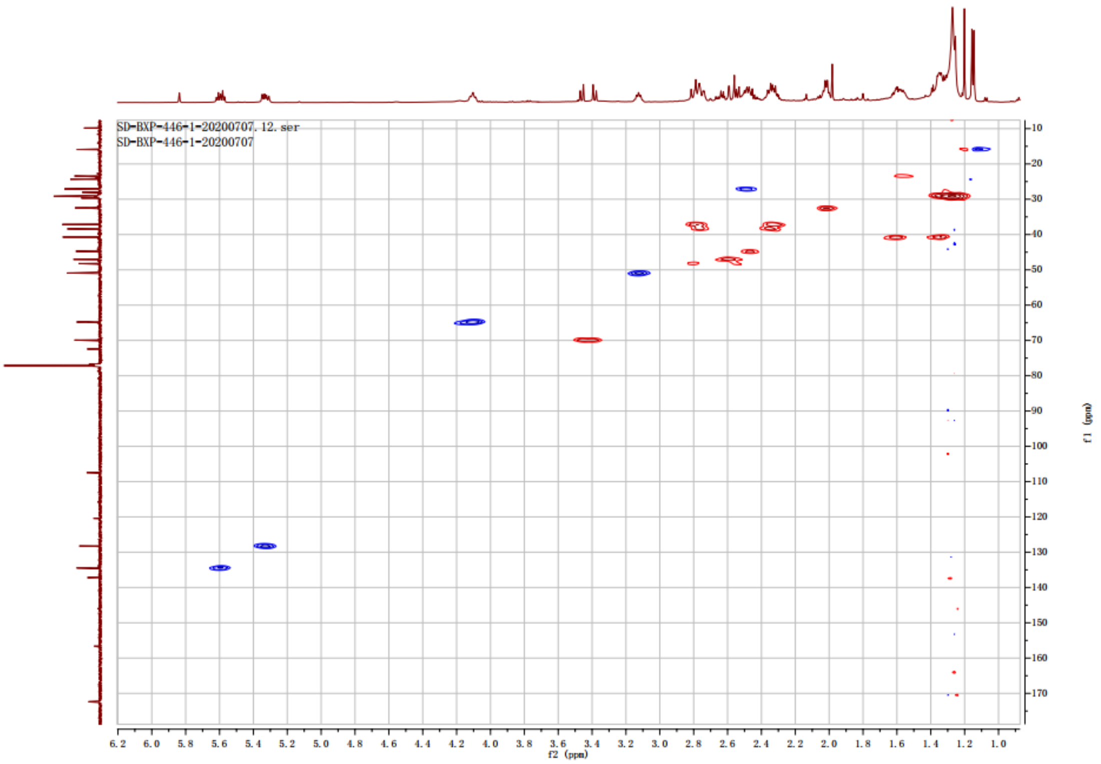

Figure S31. HSQC spectrum of gladiofungin G (**6**) in $CDCl_3$

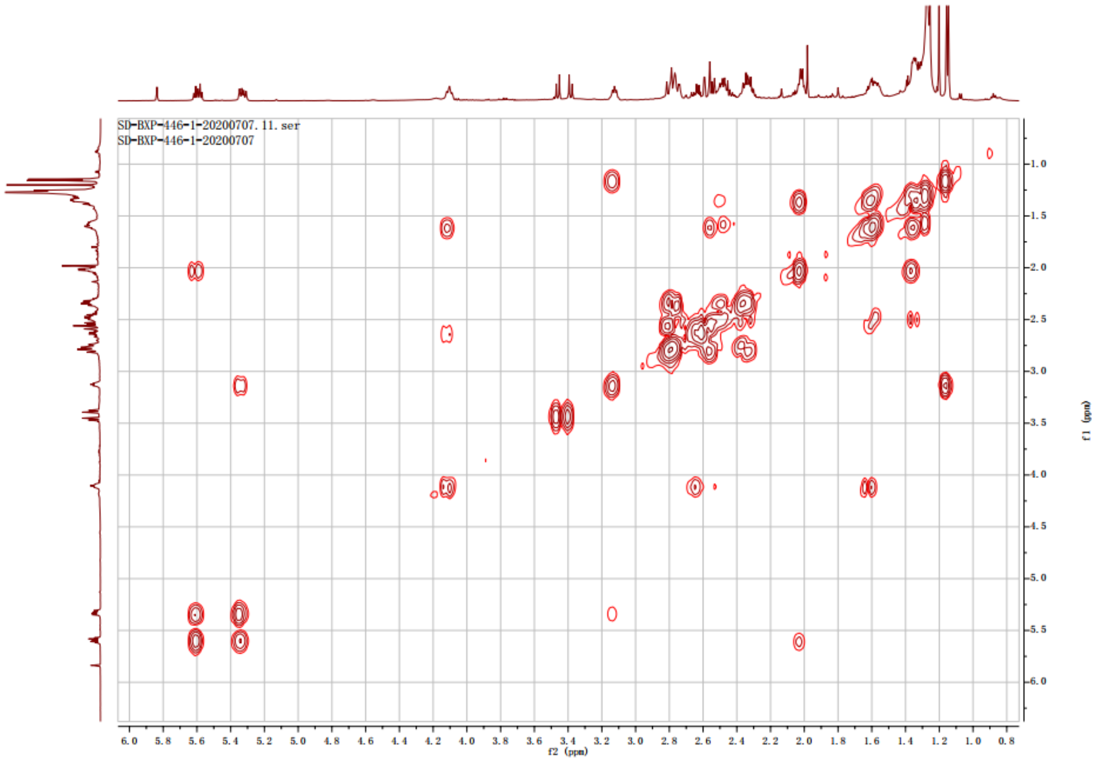

Figure S32. ^{1}H-^{1}H COSY spectrum of gladiofungin G (**6**) in $CDCl_3$

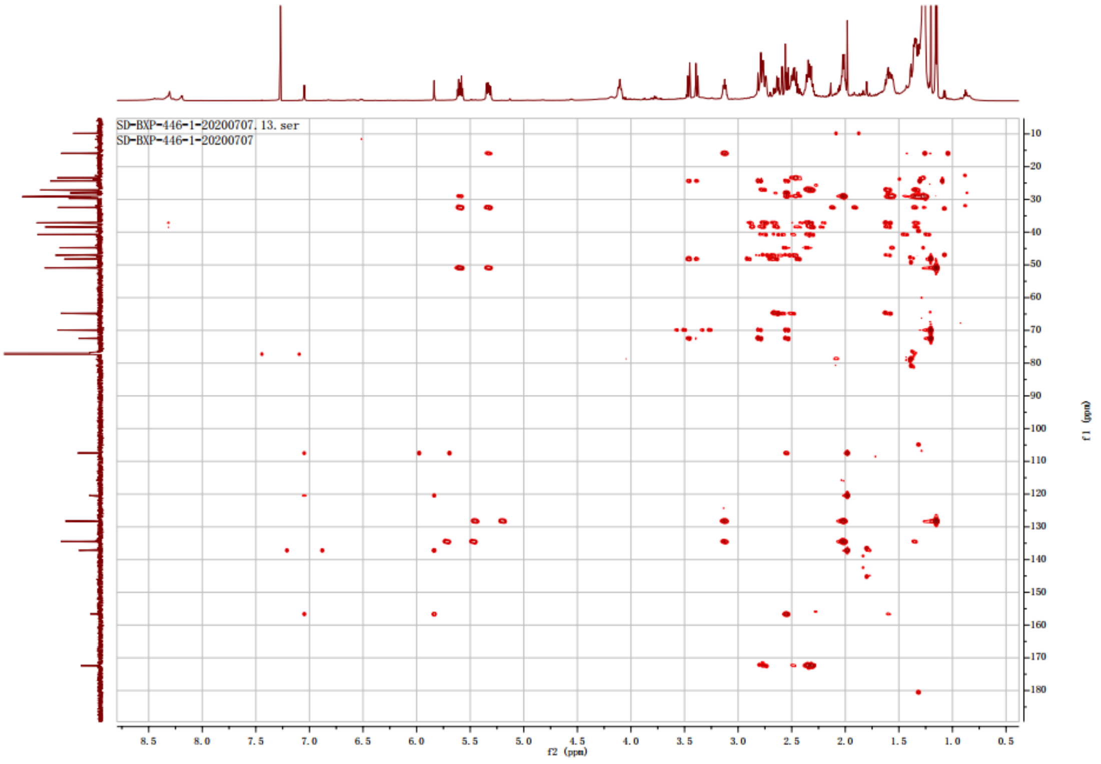

Figure S33. HMBC spectrum of gladiofungin G (**6**) in $CDCl_3$

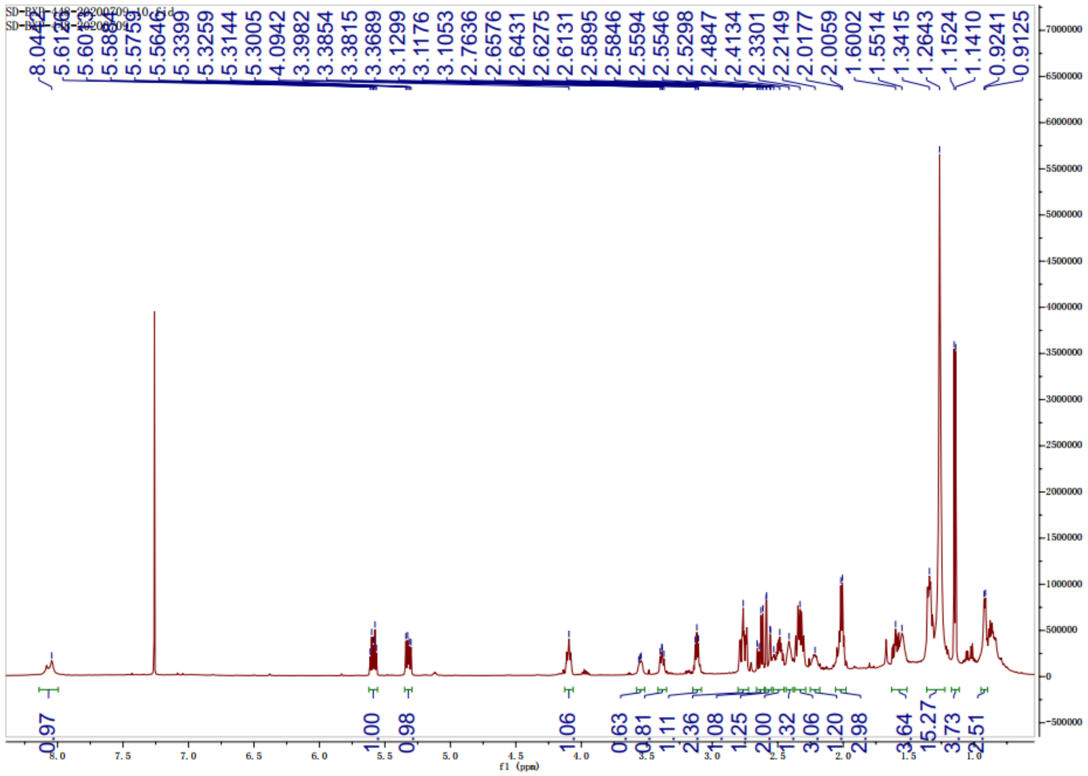

Figure S34. ^{1}H NMR spectrum of gladiofungin H (**7**) in $CDCl_3$

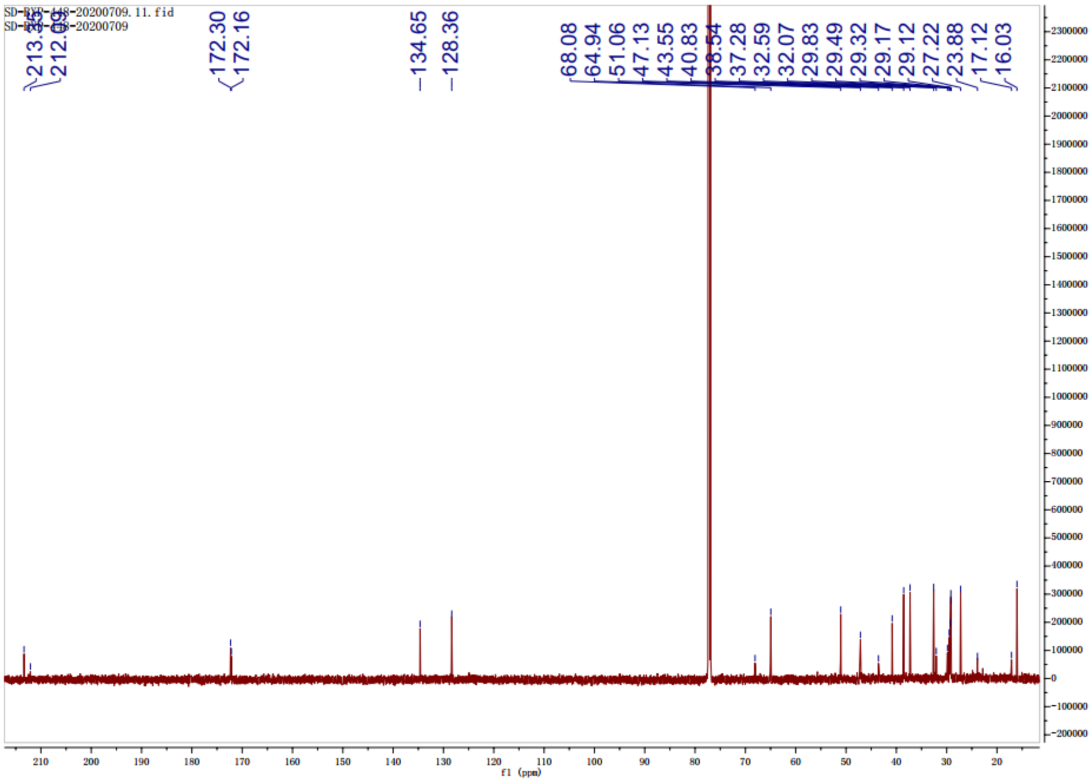

Figure S35. ^{13}C NMR spectrum of gladiofungin H (**7**) in $CDCl_3$

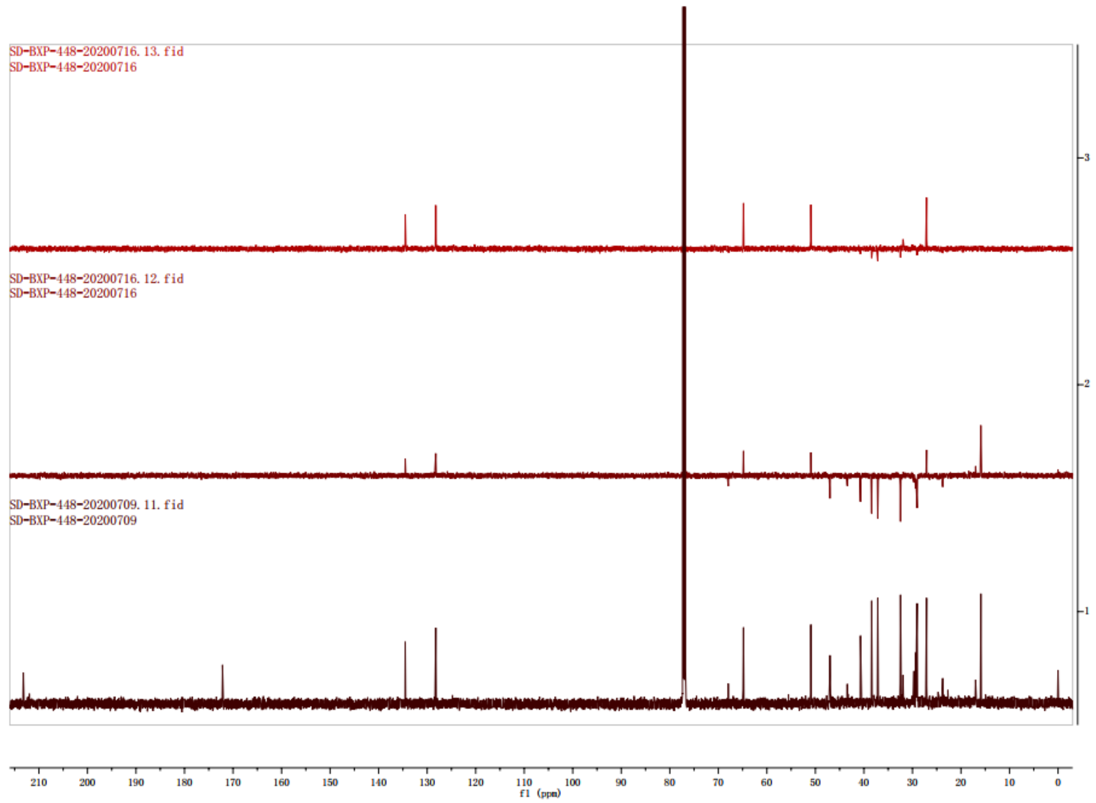

Figure S36. DEPT spectrum of gladiofungin H (**7**) in $CDCl_3$

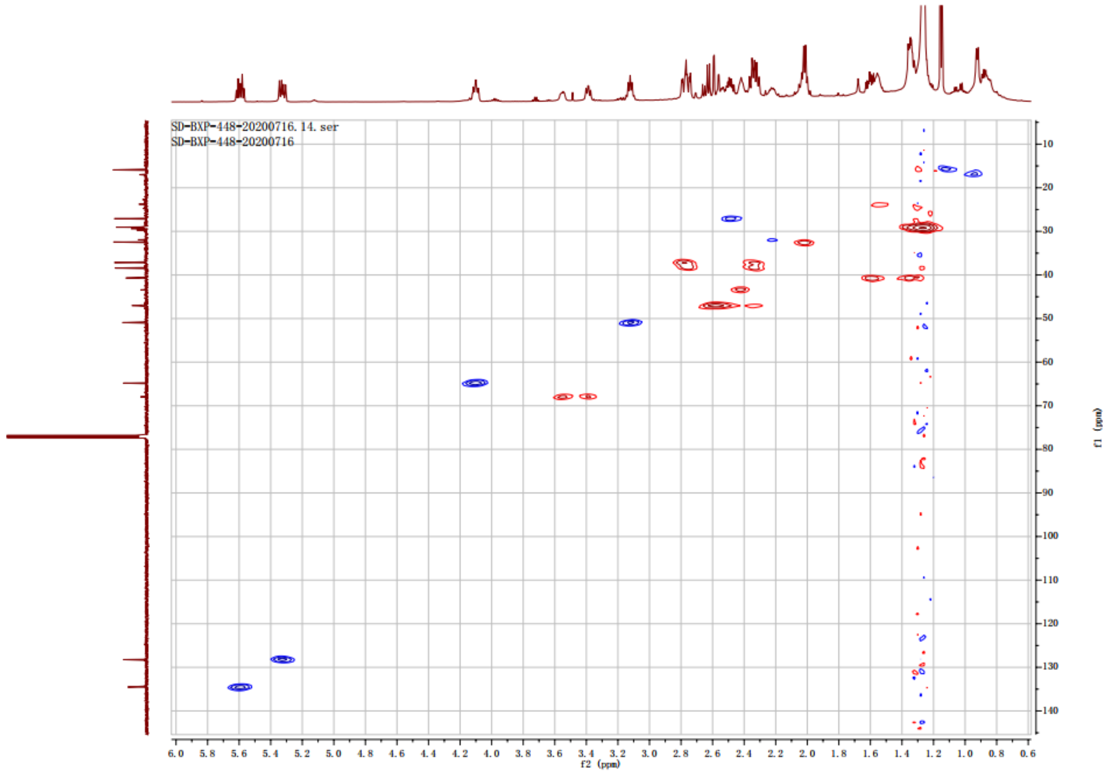

Figure S37. HSQC spectrum of gladiofungin H (**7**) in $CDCl_3$

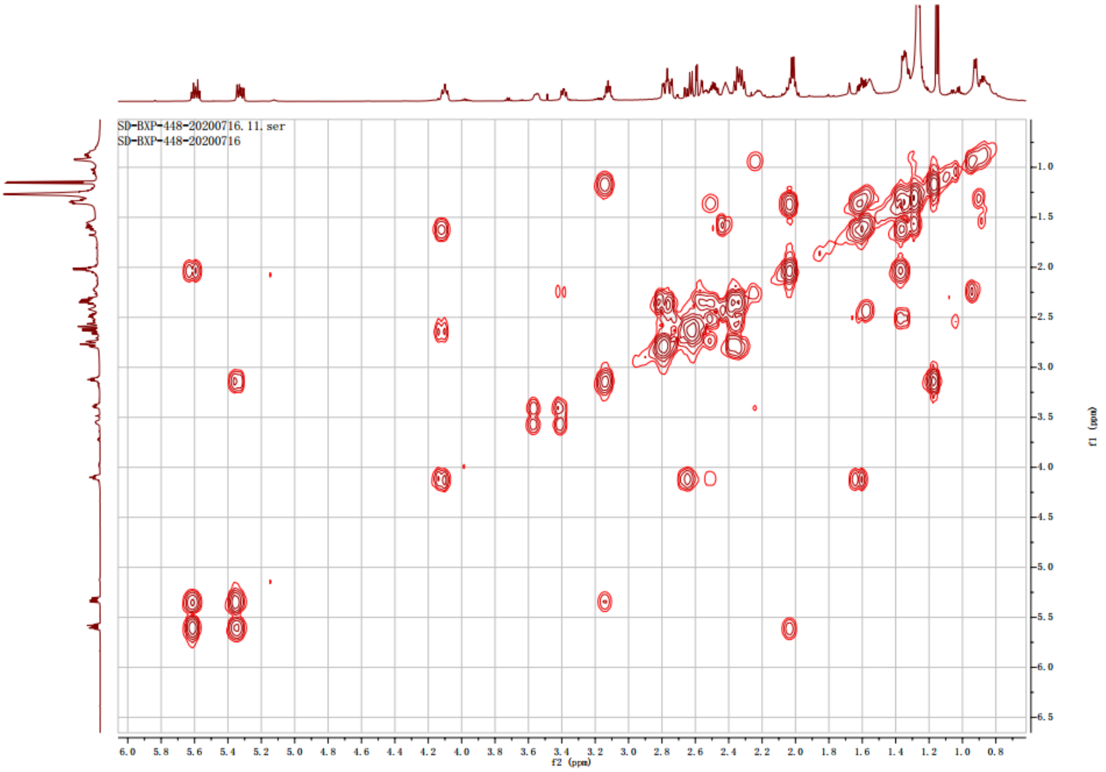

Figure S38. ^{1}H-^{1}H COSY spectrum of gladiofungin H (**7**) in $CDCl_3$

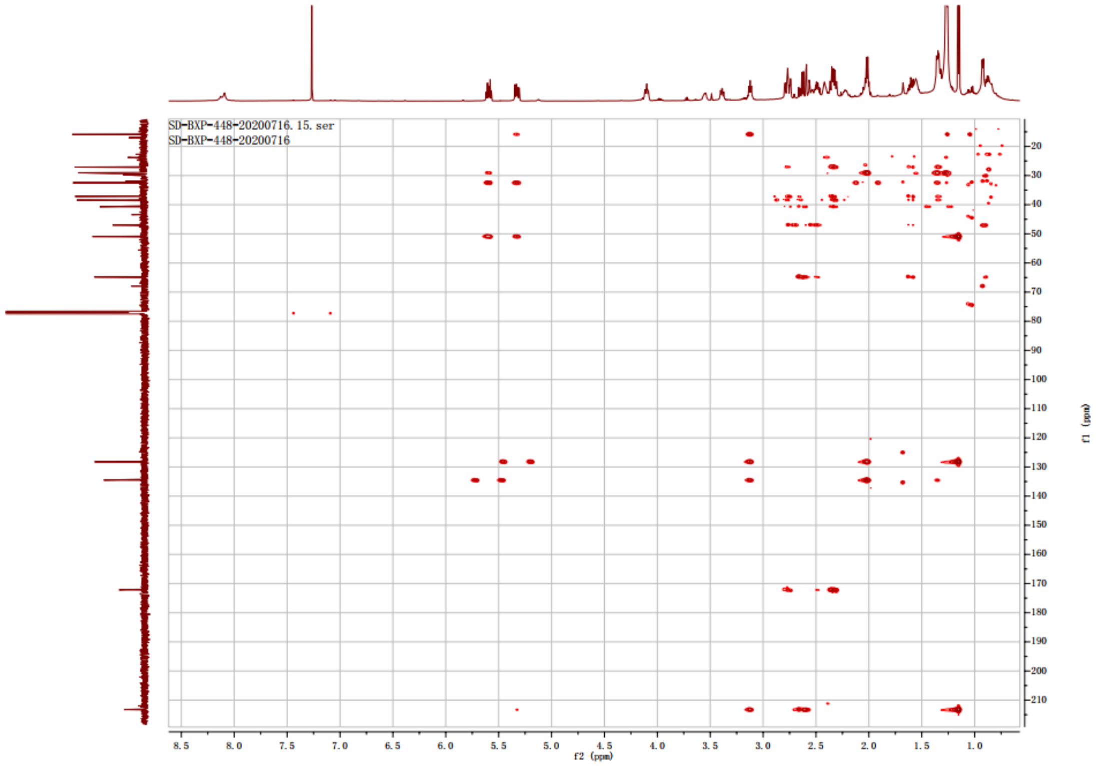

Figure S39. HMBC spectrum of gladiofungin H (**7**) in $CDCl_3$
